# Examining the links between achievement goals, students' approaches to learning, and learning outcomes in business studies

**DOI:** 10.3389/fpsyg.2026.1837226

**Published:** 2026-10-07

**Authors:** Hannu Ojala, Heta Tuominen, Markku Niemivirta, Jari Huikku

**Affiliations:** 1 Department of Accounting and Finance, University of Eastern Finland, Kuopio, Finland; 2 Department of Accounting and Business Law, Aalto University School of Business, Espoo, Finland; 3 School of Applied Educational Science and Teacher Education, University of Eastern Finland, Joensuu, Finland

**Keywords:** achievement goals, approaches to learning, business education, learning outcomes, motivation

## Abstract

This study investigated how achievement goals and students' approaches to learning were jointly associated with academic success in business education. Drawing on survey data from 195 first-semester students enrolled in an introductory accounting course at a Nordic business school, we employed latent profile analysis to identify distinct learning profiles. We identified four learning profiles: Deep–Organized, Deep-Dominated, Surface-Dominated, and Benchmark. The majority of students (~70%) belonged to the two deep-oriented profiles, whereas only a small minority (6%) relied primarily on surface strategies. We then used multinomial logistic regression to examine associations with profile membership, achievement goals and academic outcomes. Mastery and performance-approach goals were associated with a higher likelihood of belonging to deep-oriented profiles, whereas performance-avoidance goals were associated with a lower likelihood of belonging to these profiles after accounting for the other achievement goals and control variables. The Organized approach was a strong predictor of academic achievement when the three continuous approaches to learning were used as predictors without achievement goals. However, approaches to learning profiles showed limited predictive power for academic achievement when motivational orientations were controlled for. Instead, performance-approach goals emerged as a comparatively robust predictor of both proximal (course-level) and distal (longer-term academic achievement during the bachelor's programme) outcomes (retrieved from university and public records), although the strength and statistical significance of the association varied across model specifications. These findings highlight the strong association of motivational orientations, particularly performance-approach goals, with students' academic trajectories in competitive business school contexts.

## 1 Introduction

This study addresses the following central research question: How do achievement goals associate with first-year business students' approaches to learning, and to what extent do these motivational orientations and learning profiles predict both short-term and long-term academic success in accounting education? To address this question, we examine the interplay between students' motivational orientations and their approaches to learning in an introduction to accounting course. Specifically, we investigate whether distinct profiles of learning approaches emerge among first-semester business students, how achievement goals associate with membership in these profiles, and how both goals and profiles relate to proximal (course-level) and distal (top academic achievement during the bachelor's programme) academic outcomes.

This inquiry builds on prior research highlighting business and accounting students' performance-oriented and assessment-driven nature, shaped by their epistemological beliefs (Lucas and Tan, 2013). Extending this line of work, Cano and Berbén (2009) demonstrated that achievement goals and approaches to learning are systematically related: mastery-approach goals align with deep approaches, whereas performance-avoidance goals align with surface approaches, and academic outcomes differ across distinct goal profiles. Similarly, Diseth and Kobbeltvedt (2010) showed that achievement goals mediate the relationship between motivational dispositions and learning strategies, and that strategic learning strategies predict examination performance. Taken together, these findings indicate that achievement goals and learning approaches operate as intertwined mechanisms through which students' motivation shapes academic outcomes. However, such integrative analyses remain underexplored in accounting and business education, particularly with respect to longer-term academic success.

To address this gap, the present study integrates Achievement Goal Theory, the Student Approaches to Learning framework, and academic learning outcomes, employing both person-oriented (cf. Niemivirta et al., 2019) and variable-oriented analyses. Drawing on survey data from 195 first-semester business students enrolled in an introductory accounting course at a Nordic university, we examine how motivational orientations and learning approaches jointly relate to academic performance. The motivation-related data were collected from students in an Introduction to Accounting course, a core first-year subject, in response to calls for further research into the nature of student motivation within business and accounting (e.g., Lucas and Tan, 2013, 120).

### 1.1 Contribution

This study makes four specific contributions to the accounting education literature. First, it integrates Achievement Goal Theory and the Student Approaches to Learning framework within a single empirical model in the context of introductory accounting. While prior studies have primarily examined these perspectives separately, this study demonstrates that achievement goals associate with membership in empirically derived learning approach profiles and that both are associated with academic outcomes. Second, it adopts a person-oriented latent profile approach to identify distinct learning configurations among first-semester business students.

Third, the study compares the predictive power of learning profiles and achievement goals for both proximal and distal outcomes. The findings show that although adaptive learning profiles are descriptively associated with stronger academic outcomes, performance-approach goals consistently explain academic success once both are considered simultaneously. This contributes to an understanding of the motivational and learning-related factors associated with achievement in a competitive business school setting.

Fourth, by incorporating a distal indicator of top academic achievement derived from publicly available records, the study extends accounting education research beyond immediate course performance. It demonstrates that motivational orientations, especially performance-approach goals, are associated not only with examination success but also with the likelihood of being recognized as a top-performing student at the completion of the bachelor's programme.

The remainder of the paper is structured as follows. The next section reviews the theoretical background on achievement goals, approaches to learning, and their associations with academic outcomes. The subsequent section describes the research design, data, and analytical methods. We then present the empirical results, followed by a discussion of the findings, implications for business and accounting education, limitations, and directions for future research.

## 2 Literature review

### 2.1 Students' approaches to learning

The Student Approaches to Learning (SAL) framework (Biggs, 1987; Entwistle and Ramsden, 1983; Marton and Säljö, 1976) examines how students engage with academic tasks in practice. Three main approaches are identified: the deep approach to learning, involving intrinsic interest, critical thinking, and knowledge integration; the surface approach to learning, involving rote memorisation and minimal conceptual engagement; and the organized approach to learning, reflecting students' capacity to plan and manage study activities (Entwistle and McCune, 2004; Parpala et al., 2010). Importantly, students' approaches to learning are shaped by both context and motivation. High workloads, competitive cultures, and assessment formats can encourage surface approaches, whereas supportive teaching environments and meaningful tasks can promote a deep approach to learning (Struyven et al., 2005).

Consistent with this contextual sensitivity, research shows that distinct profiles of learning approaches can be detected across disciplines (Parpala et al., 2022). Parpala et al. (2022) find that the Deep-Organized profile was the most common across Law, Political Science, Psychology, Economics, Business Communication, and Business Technology, and that students in this profile report the highest self-efficacy and academic achievement. However, disciplinary environments also appear to shape how learning approaches develop over time. Gow et al. (1994) found a steep decline in deep approach during the first year of studying accounting, and Byrne and Flood (2007) suggest that business students' learning approaches are consistently pragmatic and outcome-driven, shaped by the performance-oriented nature of business school environments. Among first-year business students specifically, poor teaching quality, rather than perceived workload, drives a shift from deep to surface approaches to learning (Huikku et al., 2026).

While Huikku et al. (2026) identified first-year business students' approaches-to-learning profiles using only the Deep and Surface approach scores as inputs to the latent profile analysis, there may be grounds for incorporating the Organized approach as an additional input. Indeed, Parpala et al. (2010) examined students' approaches to learning across 10 faculties at the University of Helsinki. Although the University of Helsinki does not include a business school, the study revealed that students who scored highly on items measuring organized studying were particularly concentrated in the Faculties of Law and Veterinary Medicine. Parpala et al. (2010) interpreted this finding to suggest that law students tend to take responsibility for their own learning and studying, whereas veterinary medicine students rely more heavily on teachers and the curriculum. Given that elements of both law-like independence and school-like structure can be observed in business schools, it is plausible to argue that business students, too, might be inclined to adopt organized studying approaches.

Taken together, based on the above discussion, we form the first research question.

**RQ1**. *What types of approaches to learning profiles exist among first-semester business students?*

### 2.2 Students' achievement goals and goal orientations

Students' engagement is fundamentally shaped by their motivational orientations. A widely used framework for understanding these orientations is Achievement Goal Theory (AGT) (Ames, 1992; Dweck and Leggett, 1988; Elliot and McGregor, 2001). Within this perspective, achievement goals refer to the purposes students pursue in academic settings, that is, why they engage in learning activities and how they define success.

Research grounded in AGT distinguishes between mastery goals and performance goals (Harackiewicz et al., 1997; Pintrich, 2000). Students endorsing mastery goals strive to develop competence and deepen their understanding, whereas those pursuing performance goals aim to demonstrate competence relative to others. Performance goals are further divided into performance-approach goals, characterized by striving to outperform peers, and performance-avoidance goals, characterized by efforts to avoid failure or negative evaluation (Elliot and Church, 1997; Elliot and McGregor, 2001).

These goal orientations are associated with distinct educational outcomes. Mastery-approach goals typically support deep engagement and resilience, whereas performance-avoidance goals are consistently associated with anxiety, surface approaches to learning, and other maladaptive outcomes (Niemivirta et al., 2019). Although the 2 × 2 achievement goal framework also distinguishes mastery-avoidance goals, which involve striving to avoid failing to master or understand a task (Elliot, 1999; Elliot and McGregor, 2001), this orientation was not included in the present study, given concerns raised in prior research regarding its conceptual and empirical distinctiveness (cf. DeShon and Gillespie, 2005; p. 1109). The empirical analyses therefore focus on the three goal orientations measured in our data: mastery-approach, performance-approach, and performance-avoidance.

In business school contexts, structural features such as competitive grading and high-stakes assessment tend to reinforce performance-oriented goals (Huikku et al., 2022). Empirical evidence suggests that business students frequently exhibit strong performance-approach orientations, particularly in accounting courses where success is closely tied to numerical accuracy and examination performance (Huikku et al., 2022). With regard to gender, the literature review by Meece et al. (2006) concludes that no clear overall patterns of gender differences in achievement goal orientations emerge. However, findings from business education provide some nuance: Bråten and Strømsø (2006) report that female students show a marginally higher tendency to endorse performance-avoidance goals than male students; Tempelaar et al. (2015) find modest (or no) gender differences in mastery-approach goals; and Huikku et al. (2022) observe that first-year male students tend to adopt performance-approach goals.

Importantly, prior research demonstrates systematic links between achievement goals and students' approaches to learning. Mastery goals are associated with deep and organized approach to learning, whereas performance-approach goals are associated with surface approach (Fenollar et al., 2007) or marginally associated with it (Elliot and McGregor, 2001). Performance-avoidance goals are consistently associated with surface approach to learning (Elliot and McGregor, 2001). Studies in accounting education confirm these relationships: deep approaches foster conceptual understanding of accounting as a decision-making tool, whereas surface approaches are linked to fragmented knowledge and weaker performance on analytical tasks (Everaert et al., 2017; Lucas, 2001). Organized approaches, in turn, support both mastery and performance-approach goals by enabling students to manage study demands effectively; however, these findings are based on an analysis of construction workers' learning at work (Puhakka et al., 2025).

More recent integrative research further underscores the close interconnection between achievement goals and approaches to learning. Cano and Berbén (2009) demonstrate that mastery-oriented goals align with deep approaches, whereas performance-avoidance goals align with surface strategies. Similarly, Huikku et al. (2026) show that first-year business students' approaches-to-learning profiles, constructed using the Deep and Surface approach scores as inputs, shift from deep-dominated to surface-dominated profiles when mastery-approach goals weaken, and performance-approach goals strengthen.

Given that an organized approach to learning appears particularly relevant for first-year business students, it becomes important to extend this line of inquiry. Specifically, there is a need to examine how achievement goals associate with students' membership in approaches-to-learning profiles constructed using all three types of learning approaches: deep, surface, and organized. Accordingly, the second research question is formulated as follows:

**RQ2**. *How do achievement goals associate with students' membership in these approaches to learning profiles?*

### 2.3 Achievement goals and approaches to learning as predictors of academic success

Building on the identification of learning approach profiles (RQ1) and the examination of how achievement goals associate with students' membership in these profiles (RQ2), the next step is to consider their implications for academic success. While prior research has established links between achievement goals and approaches to learning, less is known about how these configurations jointly influence both immediate and longer-term learning outcomes within naturally occurring student profiles.

The present study addresses this gap by applying latent profile analysis (LPA) to identify naturally occurring patterns of learning approaches and by examining how different goal orientations associate with these profiles and predict subsequent academic outcomes. Evidence integrating Achievement Goal Theory (AGT) and the Student Approaches to Learning (SAL) framework suggests that combinations of achievement goals may translate into academic success by influencing learning strategies. For example, Diseth and Kobbeltvedt (2010) demonstrated that organized learning approaches mediated the relationship between achievement goals and examination grades.

More broadly, variable-oriented research has revealed structured relationships among motivational dispositions, achievement goals, learning strategies, and academic performance. In a hierarchical structural equation model, Diseth and Kobbeltvedt (2010) showed that mastery and performance-approach goals were positively associated with organized learning approaches and examination grades, whereas performance-avoidance goals and surface approaches were negatively related to achievement. Importantly, learning approaches mediated the relationship between achievement goals and academic performance, underscoring the need to examine motivational orientations and learning behaviors jointly rather than in isolation.

However, although prior research has clarified part of the relationships at the variable level, there remains a need to integrate our understanding of how motivational and learning configurations operate together regarding both proximal and distal academic outcomes. In particular, it is important to examine how profile membership (reflecting combinations of deep, surface, and organized approaches) relates to academic outcomes, and how achievement goals associate with profile membership and predict subsequent academic success. Such an approach allows for a more holistic understanding of how students' motivational orientations and learning behaviors are jointly associated with their academic trajectories.

To address this need, we integrate motivational and learning perspectives by combining variable-oriented analyses of achievement goals with person-oriented analyses of students' approaches-to-learning profiles constructed from the Deep, Surface, and Organized approach scores. We then examine how these motivational orientations and learning profiles are associated with academic success over time within a business school bachelor's programme. Accordingly, we formulate the third research question as follows:

**RQ3**. *How are approaches to learning profiles and achievement goals associated with proximal and distal learning outcomes?*

## 3 Methodology

### 3.1 Participants and context of the study

The study sample comprises 195 first-semester business students enrolled at a Finnish university in Autumn 2017. The participants were recruited from a compulsory introductory business course, which serves as a foundation for further studies in business administration and accounting. The course structure includes lectures, exercises, group work, and examinations, providing a setting where students engage in various learning strategies.

The gender distribution in the sample reflects typical enrolment patterns in business education, with 62% male and 38% female students. The participants had diverse academic backgrounds, including varying levels of prior experience in business-related subjects. The business school setting is highly structured, where students' academic performance determines their access to major selection, exchange programmes, and career opportunities, reinforcing the relevance of achievement goals and approaches to learning in shaping student success.

The business school granted ethical approval to investigate associations between students' achievement goals, study success, individual characteristics, and perceptions of the learning environment. Additionally, informed consent was obtained from each participating student through the cover page of the research questionnaire. Participation was emphasized as entirely voluntary, and those who chose to take part were invited to complete the questionnaire at the beginning of a lecture, during which teaching was briefly paused. This arrangement ensured that students did not need to use their free time outside class and did not miss any lecture content. Participants were also assured that all responses would remain confidential and that strict measures were taken to safeguard their anonymity. The distal outcome was constructed by matching participants to publicly available records of top-performing students; the matching was used solely for analytical purposes, and no names or other student-level identifying information are reported.

### 3.2 Measures

#### 3.2.1 Achievement goals

Students' achievement goal orientations were assessed using an adapted version of the Achievement Goal Questionnaire-Revised (AGQ-R; Elliot and Murayama, 2008). The AGQ-R builds on the 2 × 2 achievement goal framework and the original Achievement Goal Questionnaire (AGQ) developed by Elliot and McGregor (2001). The 2 × 2 framework distinguishes achievement goals along two dimensions: the definition of competence (mastery vs. performance) and its valence (approach vs. avoidance). Their combination yields four theoretically distinct goal orientations: mastery-approach, mastery-avoidance, performance-approach, and performance-avoidance (Elliot and McGregor, 2001). Elliot and Murayama (2008) subsequently revised the original AGQ to improve the conceptual correspondence between achievement goals and their measurement, resulting in the AGQ-R.

The present study focused on three of these orientations: mastery-approach, performance-approach, and performance-avoidance. Each orientation was assessed with three items adapted from the AGQ-R. Mastery-approach goals reflect striving to develop competence and achieve a thorough understanding of the task; performance-approach goals reflect striving to perform well relative to others; and performance-avoidance goals reflect striving to avoid performing worse than others. Mastery-avoidance was not included in the measurement used in the present study and was therefore not included in the empirical analyses. The AGQ-R items were translated into Finnish and adapted to the introductory accounting course context while retaining the substantive content of the original items.

Responses to the nine achievement-goal items were provided on a five-point Likert scale ranging from 1 (strongly disagree) to 5 (strongly agree). The three items corresponding to each orientation were combined to form the Mastery-approach, Performance-approach, and Performance-avoidance scales, respectively. The complete wording of the measurement items is reported in Table 1 in the Section 4.

Table 1Confirmatory factor analysis, reliability, convergent validity, and latent-factor correlations for the approaches-to-learning and achievement goal scales (*n* = 195).

**Table:** 

Latent factor	Measurement item	Standardized loading	SE	*p*
Panel A. Confirmatory factor analysis of the approaches-to-learning scales				
**Deep approach**	Ideas I came across in my course reading often set me off on long chains of thought.	0.567	0.070	< 0.001
	I looked at the evidence carefully to reach my own conclusion about what I was studying.	0.560	0.069	< 0.001
	I tried to relate new material, as I was reading it, to what I already knew on that topic.	0.631	0.061	< 0.001
	I tried to relate what I had learned in this course to what I had learned in other courses.	0.740	0.060	< 0.001
**Surface approach**	I often had trouble making sense of the things I had to remember.	0.694	0.069	< 0.001
	Much of what I learned seemed no more than lots of unrelated bits and pieces in my mind.	0.432	0.076	< 0.001
	Topics were presented in such complicated ways that I often could not see what was meant.	0.681	0.068	< 0.001
	Often, I had to learn over and over things that did not really make much sense to me.	0.427	0.075	< 0.001
Organized approach	I generally put a lot of effort into my studying.	0.481	0.075	< 0.001
	On the whole, I was quite systematic and organized in my studying.	0.661	0.071	< 0.001
	I organized my study time carefully to make the best use of it.	0.584	0.074	< 0.001
	I made a plan to ensure that I covered the entire curriculum during the scheduled timetable.	0.556	0.071	< 0.001
**Scale**	**Number of items**	**Cronbach's α**	**CR**	**AVE**
Panel B. Reliability and convergent-validity statistics				
Deep approach	4	0.716	0.720	0.395
Surface approach	4	0.628	0.651	0.329
Organized approach	4	0.653	0.660	0.330

**Table:** 

Factor pair	Correlation	SE	*p*
Panel C. Latent-factor correlations and overall CFA model fit			
C1. Correlations among the latent factors			
Deep approach–Surface approach	−0.352	0.093	< 0.001
Deep approach–Organized approach	0.306	0.099	0.002
Surface approach–Organized approach	−0.280	0.105	0.008

**Table:** 

Fit statistic	Value
C2. Overall fit of the correlated three-factor CFA model	
Observations	195
Model chi-square	χ^2^ (51)=112.332, *p* < 0.001
Comparative fit index	CFI = 0.852
Tucker–Lewis index	TLI = 0.808
Root mean square error of approximation	RMSEA = 0.079, 90% CI (0.059, 0.098)
Test of close fit	p_close = 0.010
Standardized root mean squared residual	SRMR = 0.072
Coefficient of determination	CD = 0.970

**Table:** 

Latent factor	Measurement item	Standardized loading	SE	*p*
Panel D. Confirmatory factor analysis of the achievement-goal scales				
**Mastery-approach**	My aim is to completely master the material presented in this class.	0.780	0.059	< 0.001
	My goal is to learn as much as possible.	0.601	0.065	< 0.001
	I am striving to understand the content of this course as thoroughly as possible.	0.700	0.060	< 0.001
**Performance-approach**	I am striving to do well compared to other students.	0.919	0.014	< 0.001
	My aim is to perform well relative to other students.	0.923	0.014	< 0.001
	My goal is to perform better than the other students.	0.949	0.011	< 0.001
**Performance-avoidance**	My goal is to avoid performing poorly compared to others.	0.782	0.031	< 0.001
	I am striving to avoid performing worse than others.	0.956	0.017	< 0.001
	My aim is to avoid doing worse than other students.	0.845	0.025	< 0.001
**Scale**	**Number of items**	**Cronbach's α**	**CR**	**AVE**
Panel E. Reliability and convergent-validity statistics				
Mastery-approach	3	0.736	0.738	0.487
Performance-approach	3	0.951	0.951	0.865
Performance-avoidance	3	0.890	0.898	0.747

**Table:** 

Factor pair	Correlation	SE	*p*
Panel F. Latent-factor correlations and overall CFA model fit			
C1. Correlations among the latent factors			
Mastery-approach–Performance-approach	0.511	0.071	< 0.001
Mastery-approach–Performance-avoidance	0.459	0.072	< 0.001
Performance-approach–Performance-avoidance	0.646	0.047	< 0.001

**Table:** 

Fit statistic	Value
C2. Overall fit of the correlated three-factor CFA model	
Observations	195
Model chi-square	χ^2^ (24) = 49.156, *p* = 0.002
Comparative fit index	CFI = 0.980
Tucker–Lewis index	TLI = 0.969
Root mean square error of approximation	RMSEA = 0.074, 90% CI (0.044, 0.103)
Test of close fit	p_close = 0.091
Standardized root mean squared residual	SRMR = 0.052
Coefficient of determination	CD = 0.999

*Panels* ***A*** *and* ***B***. The confirmatory factor analysis specified three correlated latent factors: Deep, Surface, and Organized approaches to learning. Parameters were estimated using maximum likelihood with standard errors based on the observed information matrix. Standard errors and *p*-values for the standardized loadings were calculated using the delta method. Values reported by Stata as *p* = 0.000 are presented as *p* < 0.001.

Consistent with the data-preparation procedure used in the original analyses, missing item-level responses were replaced with the mean of the corresponding item before estimation. Among the approaches-to-learning indicators included in the CFA, this procedure concerned one missing response to an organized-learning item.

CR, composite reliability; AVE, average variance extracted.

*Panel C*. The latent-factor variances were fixed at one. Consequently, the reported latent covariances are numerically equivalent to latent-factor correlations. Their standard errors and *p*-values were calculated using the delta method.

CFI, comparative fit index; TLI, Tucker–Lewis index; RMSEA, root mean square error of approximation; CI, confidence interval; p_close, probability associated with the test of close fit; SRMR, standardized root mean squared residual; CD, coefficient of determination.

*Panels* ***D*** *and* ***E***. The confirmatory factor analysis specified three correlated latent achievement-goal factors: Mastery-approach, Performance-approach, and Performance-avoidance. Each factor was measured using three items adapted from the Achievement Goal Questionnaire–Revised (AGQ-R; Elliot and Murayama, 2008). Responses were provided on a five-point scale ranging from 1 (totally disagree) to 5 (totally agree).

Parameters were estimated using maximum likelihood with standard errors based on the observed information matrix. Standard errors and p-values for the standardized loadings were calculated using the delta method. Values reported by Stata as *p* = 0.000 are presented as *p* < 0.001.

Consistent with the data-preparation procedure used in the original analyses, missing item-level responses were replaced with the mean of the corresponding item before estimation.

CR, composite reliability; AVE, average variance extracted.

*Panel* ***F***. The latent-factor variances were fixed at one. Consequently, the reported latent covariances are numerically equivalent to latent-factor correlations. Their standard errors and *p*-values were calculated using the delta method.

CFI, comparative fit index; TLI, Tucker–Lewis index; RMSEA, root mean square error of approximation; CI, confidence interval; p_close, probability associated with the test of close fit; SRMR, standardized root mean squared residual; CD, coefficient of determination.

#### 3.2.2 Approaches to learning

The LEARN questionnaire assessed students' approaches to learning (Parpala and Lindblom-Ylänne, 2012). The LEARN questionnaire, developed at the University of Helsinki, is a validated, research-based instrument designed to assess and enhance teaching and learning quality in higher education. Adapted from the Experiences of Teaching and Learning Questionnaire (ETLQ), it focuses on students' approaches to learning and their perceptions of the teaching-learning environment. The Finnish version integrates a modified Approaches to Learning and Studying Inventory (ALSI) (Entwistle and McCune, 2004) with additional sections on study-related challenges, workload and stress, self-efficacy, and the development of generic skills. LEARN appears relatively reliable as the reliabilities concerning the scales measuring approaches to learning vary from 0.720 to 0.797 (Parpala and Lindblom-Ylänne, 2012, 318–319).

LEARN captures three primary approaches to learning: (1) the Deep approach (e.g., “I try to find connections between different concepts”); (2) the Surface approach (e.g., “I memorize facts without trying to understand their meaning”); and (3) the Organized approach (e.g., “I plan my study time carefully”). Responses were rated on a five-point Likert scale. Mean scores were calculated for each approach, producing the continuous Deep approach, Surface approach, and Organized approach measures. These three continuous measures were used as inputs to the latent profile analysis and were also used in the additional variable-oriented analyses.

#### 3.2.3 Academic outcomes

To assess students' proximal and distal learning outcomes, we included the proximal outcome using the course total points of the Introduction to Accounting course (maximum 106 points), consisting of the final course exam (max. 60 points), two open-book mid-term exams (max. 20 points) and teamwork points (max. 26 points) to indicate immediate academic performance. The distal outcome was designed to capture high academic achievement during the bachelor's programme. The business school publicly reports the names of students recognized for achieving the highest academic performance at the completion of their bachelor's studies. We matched these publicly available records to the students participating in the study and constructed a binary indicator coded 1 for students identified in the public records as top-performing students and 0 otherwise. In total, 34 students (17.4 % of the sample) were identified as top-performing students and coded as 1. Importantly, this variable does not measure degree completion and was not constructed by dichotomising individual-level continuous GPA data. The full cumulative GPA scores of the students were not available from the public source and therefore could not be used as a continuous distal outcome. No student-level information obtained through this matching procedure is reported in the study.

### 3.3 Data analyses

The study employed a three-phase approach to examine how first-semester business students' achievement goals were associated with their learning approaches and academic outcomes, as illustrated in Figure 1.

**Figure 1:**
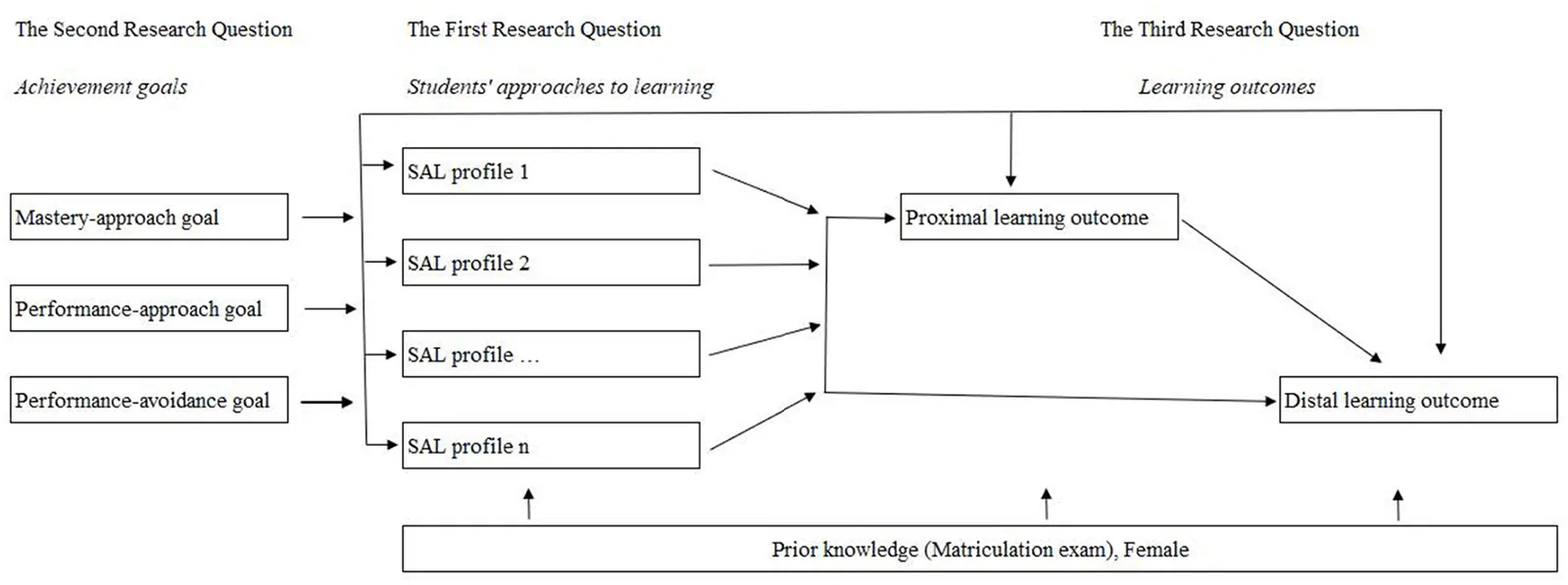
Theoretical model of the study.

In the first phase, Latent Profile Analysis (LPA) was used to identify distinct learning profiles based on students' responses to the LEARN questionnaire (Parpala and Lindblom-Ylänne, 2012), which measures the Deep, Surface, and Organized approaches to learning. The analysis was conducted in RStudio with the TidyLPA package. LPA is a model-based person-oriented analytical approach that identifies unobserved subgroups based on individuals' response patterns across continuous indicators and estimates their probabilities of profile membership (Collins and Lanza, 2013). Before conducting the latent profile analysis, we examined the measurement structure of the approaches-to-learning instrument using confirmatory factor analysis. A correlated three-factor model was specified, comprising Deep, Surface, and Organized approaches to learning, with four indicators loading on each factor. The model was estimated using maximum likelihood with standard errors based on the observed information matrix. Consistent with the data-preparation procedure used in the original analyses, missing item-level responses were replaced with the mean of the corresponding item before estimation. Among the approaches-to-learning indicators, this procedure concerned one missing response to an organized-learning item. Model fit was evaluated using the chi-square statistic, CFI, TLI, RMSEA with its 90% confidence interval and test of close fit, and SRMR. Standardized factor loadings, latent-factor correlations, Cronbach's alpha, composite reliability, and average variance extracted were also examined.

For the subsequent regression analyses, students were assigned to the profile for which they had the highest posterior membership probability (modal class assignment). Thus, profile membership was treated as an observed categorical variable in the subsequent analyses. This approach does not explicitly account for uncertainty in latent-profile classification. Classification quality was therefore evaluated using entropy and class-specific average posterior probabilities, which are reported in Table 2.

Table 2Latent profile model selection.

**Table:** 

Profiles	LogLik	AIC	BIC	SABIC	Entropy	BLRT	*p*	AvePP range	Smallest profile (%)
Panel A. Fit statistics for the competing profile solutions									
2	−577.27	1,174.54	**1,207.27**	1,175.60	0.545	12.68	0.010	—	31.3
3	−569.59	1,167.17	1,213.00	1,168.65	0.590	15.36	0.010	—	14.9
**4**	–**562.07**	**1,160.14**	1,219.06	**1,162.04**	**0.685**	**15.03**	**0.030**	**0.771–0.865**	**6.2**
5	−561.23	1,166.47	1,238.47	1,168.78	0.624	1.67	0.871	0.242–0.886	4.6

**Table:** 

Profile	*n*	%	AvePP	Deep	Surface	Organized
Panel B. Classification results for the retained four-profile solution						
Deep–Organized	72	36.9	0.826	4.11	1.82	3.99
Deep-Dominated	65	33.3	0.865	3.75	1.99	2.64
Benchmark	46	23.6	0.771	3.73	2.83	3.58
Surface-Dominated	12	6.2	0.863	3.06	3.56	2.75

LogLik, log likelihood; AIC, Akaike information criterion; BIC, Bayesian information criterion; SABIC, sample-size adjusted Bayesian information criterion; BLRT, bootstrap likelihood ratio test; AvePP, average posterior probability of membership in the profile to which a student was assigned.

Lower AIC, BIC, and SABIC values indicate better model fit. The BLRT compares a model containing k profiles with a model containing k−1 profiles. A statistically significant BLRT indicates that the model containing k profiles provides a better fit than the model with one fewer profile. Entropy ranges from 0 to 1, with higher values indicating greater classification certainty. Bold values identify the retained four-profile model and the lowest value for each information criterion. BIC favored the two-profile solution, whereas AIC and SABIC favored the four-profile solution. Deep, Surface, and Organized refer to the continuous Deep approach, Surface approach, and Organized approach scores, respectively.

In the second phase, multinomial logistic regression was used to examine how achievement goals associate with membership in the approaches to learning profiles. Goals were assessed using an adapted version of the Achievement Goal Questionnaire-Revised (AGQ-R; Elliot and Murayama, 2008) and included mastery-approach, performance-approach, and performance-avoidance orientations. Gender and matriculation exam points were controlled for. Given the relatively strong association between performance-approach and performance-avoidance goals, multicollinearity among the predictors was assessed using variance inflation factors (VIFs) and tolerance statistics.

In the third phase, the associations between learning profiles and academic outcomes were examined. The proximal outcome was measured by course total points (0–106) obtained from official university records, and the distal outcome was a binary indicator of top academic achievement during the bachelor's programme (1 = identified among the publicly reported top-performing students; 0 = otherwise). Ordinary least squares regression was used to examine associations with course performance, and binary logistic regression was employed to examine associations with the distal binary outcome indicating whether a student was identified as a top-performing student at the completion of the bachelor's programme.

## 4 Empirical results

### 4.1 Empirical results regarding RQ1: the types of approaches to learning profiles

Before conducting the latent profile analysis (LPA), the psychometric properties of the three learning-approach scales (Deep, Surface, and Organized) were examined using confirmatory factor analysis and the LEARN questionnaire (Parpala and Lindblom-Ylänne, 2012).

Table 1 presents the measurement analyses for the two sets of constructs used in the study: the three approaches-to-learning scales (Panels **A–C**) and the three achievement-goal scales (Panels **D–F**). We first consider the measurement properties of the approaches-to-learning scales used to construct the latent profiles. All indicators loaded significantly on their intended factors (*p* < 0.001), with standardized loadings ranging from 0.427 to 0.740. Model fit was mixed, χ2 (51) = 112.332, *p* < 0.001, CFI = 0.852, TLI = 0.808, RMSEA = 0.079, 90% CI (0.059, 0.098), and SRMR =0.072. Although the RMSEA and SRMR suggested approximately acceptable residual fit, the CFI and TLI remained below commonly used benchmarks, indicating only partial support for the three-factor structure. The latent-factor correlations were moderate and theoretically consistent: the Deep approach was negatively related to the Surface approach (*r* = −0.352) and positively related to the Organized approach (*r* = 0.306), while the Surface and Organized approaches were negatively related (*r* = −0.280). Reliability was satisfactory for the Deep approach (α = 0.716, CR = 0.720) and more modest for the Surface approach (α = 0.628, CR = 0.651) and Organized approach (α = 0.653, CR = 0.660). AVE values ranged from 0.329 to 0.395, indicating limited convergent validity. Consequently, the three scales were retained on theoretical grounds and because all items loaded significantly on their intended factors, but results involving the Surface and Organized approaches should be interpreted cautiously.

The measurement properties of the achievement-goal scales are reported in Panels **D–F** of Table 1. All items loaded significantly, and generally strongly, on their intended factors, with standardized loadings ranging from 0.601 to 0.956 (all *p* < 0.001). Internal consistency was acceptable for Mastery-approach (α = 0.736, CR = 0.738) and high for Performance-approach (α = 0.951, CR = 0.951) and Performance-avoidance (α = 0.890, CR = 0.898). AVE exceeded the conventional 0.50 benchmark for the two performance-goal scales (0.865 and 0.747, respectively), while the Mastery-approach AVE (0.487) was marginally below this benchmark. The correlated three-factor CFA showed good overall fit, χ^2^ (24) = 49.156, *p* = 0.002, CFI = 0.980, TLI = 0.969, RMSEA = 0.074, 90% CI (0.044, 0.103), and SRMR = 0.052. The latent-factor correlations were positive and moderate to moderately strong (*r* = 0.459–0.646), indicating that the three achievement-goal orientations were related but empirically distinguishable. These results provide support for the reliability and factorial validity of the three achievement-goal scales used in the subsequent analyses.

The first research question examined whether distinct approaches-to-learning profiles could be identified among first-semester business students. Latent profile models containing two to five profiles were estimated using students' continuous Deep approach, Surface approach, and Organized approach scores. Model selection was based on multiple complementary statistical and substantive criteria rather than a single fit index, consistent with current recommendations for mixture modeling (e.g., Nylund et al., 2007; Marsh et al., 2009; Morin et al., 2011). Tein et al. (2013) note that there is no common standard for determining the best-fitting model and that researchers typically use a combination of fit criteria when determining the number of latent classes. Specifically, model fit was evaluated using the Akaike Information Criterion (AIC), Bayesian Information Criterion (BIC), sample-size adjusted Bayesian Information Criterion (SABIC), bootstrap likelihood ratio test (BLRT), entropy, posterior classification probabilities, profile sizes, and the substantive interpretability of the resulting profiles.

Table 2 presents the model fit statistics for the alternative profile solutions. Model selection was based on the combined statistical and substantive criteria described above.

Panel **A** of Table 2 provides the basis for interpreting the model-selection results. The information criteria did not unanimously identify the same profile solution. The AIC and SABIC reached their lowest values for the four-profile model, whereas the BIC favored the more parsimonious two-profile model. The BLRT indicated that the four-profile model provided a statistically significant improvement over the three-profile model (BLRT = 15.03, *p* = 0.030). In contrast, adding a fifth profile did not improve model fit (BLRT = 1.67, *p* = 0.871). The four-profile solution also produced the highest entropy (0.685) among the estimated models. Although the entropy value indicates some uncertainty in individual profile assignment, the class-specific average posterior probabilities suggest generally acceptable classification quality. The class-specific average posterior probabilities ranged from 0.771 to 0.865. The Benchmark profile had the lowest average posterior probability (0.771), indicating somewhat greater overlap with the other profiles, whereas the remaining profiles had average posterior probabilities exceeding 0.82. While Parpala et al. (2022) identified a five-profile solution, the five-profile solution was not retained in the current study because it did not provide a statistically significant improvement over the four-profile model. It also produced a lower entropy value, a very low minimum average posterior probability, and the smallest profile containing only 4.6% of the sample.

Panel **B** of Table 2 helps to interpret the retained profiles. The retained solution consisted of four conceptually distinct profiles. The Deep-Organized profile was the largest, comprising 72 students (36.9 %). These students reported high levels of the Deep and Organized approaches together with a low score on the Surface approach. The Deep-Dominated profile included 65 students (33.3 %) and was characterized by a relatively high Deep approach score but a substantially lower Organized approach score. The Benchmark profile comprised 46 students (23.6 %) and showed values relatively close to the sample averages across the three learning dimensions. The Surface-Dominated profile was the smallest, comprising 12 students (6.2 %). It was characterized by the highest Surface approach score and comparatively lower Deep and Organized approach scores. Despite its small size, its average posterior probability was 0.863, suggesting that it represented a reasonably well-defined profile. Nevertheless, results concerning this subgroup should be interpreted cautiously because of the limited number of observations.

Table 3 presents descriptive statistics for achievement goals, demographic variables, and academic outcomes across the four profiles. Clear descriptive differences emerged. Students in the Deep–Organized profile reported the highest levels of mastery-approach (mean = 4.57) and performance-approach goals (mean = 4.10), whereas students in the Surface-Dominated profile reported the lowest levels of both (mean = 3.86 and mean = 2.56, respectively). Performance-avoidance goals were also highest in the Deep–Organized profile (mean = 4.22) and lowest in the Surface-Dominated profile (mean = 3.25). The Deep–Organized students also achieved the highest average course scores and had the highest proportion of students subsequently identified as top-performing students at the completion of the bachelor's programme (distal), whereas the Surface-Dominated profile showed the weakest academic outcomes (proximal and distal). The Benchmark and Deep-Dominated profiles generally occupied intermediate positions on these variables.

**Table 3:** Descriptive statistics by approaches-to-learning profiles.

Variable	Deep-Organized			Deep-Dominated			Benchmark			Surface-Dominated		
	Mean	Median	SD	Mean	Median	SD	Mean	Median	SD	Mean	Median	SD
Deep approach	4.106	4.25	0.524	3.765	3.75	0.611	3.728	3.75	0.540	3.062	3.25	0.650
Surface approach	1.810	1.75	0.352	1.992	2.00	0.362	2.832	2.75	0.294	3.562	3.50	0.441
Organized approach	4.000	4.00	0.423	2.652	2.75	0.436	3.582	3.50	0.466	2.750	2.88	0.622
Mastery approach	4.568	4.67	0.427	4.187	4.00	0.576	4.290	4.33	0.500	3.861	4.00	0.627
Performance approach	4.103	4.00	0.888	3.687	4.00	0.961	3.274	3.00	0.900	2.556	2.50	1.057
Performance avoidance	4.221	4.33	0.946	3.914	4.00	0.899	4.101	4.00	0.807	3.250	3.67	1.272
Matriculation points	27.72	31.0	10.51	25.12	28.0	10.73	24.85	29.0	11.29	25.83	31.0	11.22
Female	0.380	0.00	0.489	0.227	0.00	0.422	0.543	1.00	0.504	0.667	1.00	0.492
Proximal	83.18	88.0	17.79	77.32	83.0	19.81	79.33	84.0	17.19	74.25	71.0	9.037
Distal	0.239	0.00	0.430	0.152	0.00	0.361	0.152	0.00	0.363	0.000	0.00	0.000

From Table 3, we can also see that regarding proximal outcomes (course total points), Deep–Organized students achieved the highest mean (83.18), while the Surface-Dominated profile reported the lowest (74.25). Distal outcomes further reinforce this descriptive pattern: 24% of Deep–Organized students were subsequently identified as top-performing students at the completion of the bachelor's programme, compared with none in the Surface-Dominated group.

Students in the Deep–Organized profile entered with the highest average matriculation points (27.72), whereas the Benchmark and Deep-Dominated groups scored lower (24.85 and 25.12, respectively). Gender composition also varied. Women were overrepresented in the Surface-Dominated (67%) and Benchmark (54%) profiles, whereas men represented a larger proportion of the Deep-Dominated group (77%).

The Benchmark profile serves as the reference category in the subsequent multinomial logistic regression analyses. To assess whether the other three profiles differ from the Benchmark profile on the continuous variables used as inputs to the latent profile analysis, Table 4 compares mean scores for the Deep approach, Surface approach, and Organized approach. Statistically significant mean differences provide additional evidence that the profiles represent substantively distinct configurations of approaches to learning.

**Table 4:** Summary of pairwise mean tests (Benchmark vs. other SAL profiles).

Variable	Comparison profile	Benchmark profile	Comparison profile	Difference	*p-value*
Deep approach	Deep-Organized	3.728	4.106	0.378^***^	0.001
Deep approach	Surface-Dominated	3.728	3.062	−0.666^***^	0.001
Deep approach	Deep-Dominated	3.728	3.765	0.037	0.743
Surface approach	Deep-Organized	2.832	1.810	−1.022^***^	< 0.001
Surface approach	Surface-Dominated	2.832	3.562	0.731^***^	< 0.001
Surface approach	Deep-Dominated	2.832	1.992	−0.839^***^	< 0.001
Organized approach	Deep-Organized	3.582	4.000	0.418^***^	< 0.001
Organized approach	Surface-Dominated	3.582	2.750	−0.832^***^	< 0.001
Organized approach	Deep-Dominated	3.582	2.651	−0.930^***^	< 0.001

^***^p < 0.01.

In substantive terms, the four profiles represent different combinations of learning strategies. Deep–Organized students combine relatively strong meaning-oriented learning with systematic organization and low reliance on surface strategies. Deep-Dominated students similarly emphasize deep learning and report relatively little surface learning, but are considerably less organized. Surface-Dominated students show the opposite pattern, with relatively high reliance on surface strategies and comparatively low levels of deep and organized learning. The Benchmark profile represents a more balanced configuration, without the pronounced strengths or weaknesses characterizing the other three profiles. These configurations are illustrated in Figure 2.

**Figure 2:**
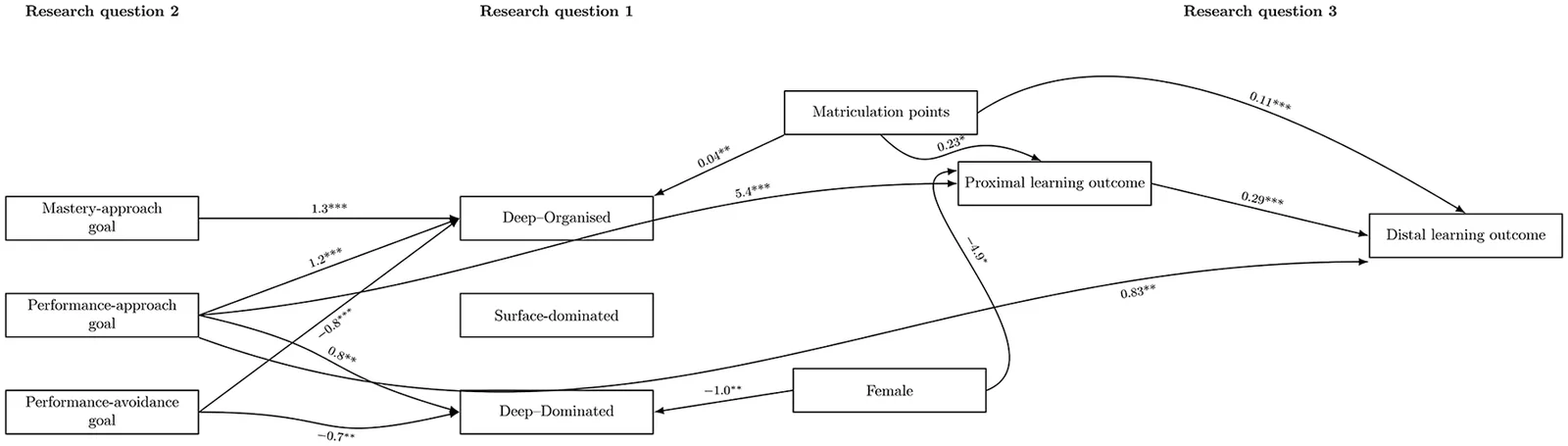
Summary of the main empirical findings. Slope coefficients have been presented for associations if *p* < 0.10. ^***^p < 0.01, ^**^p < 0.05, ^*^p < 0.10.

To further evaluate whether the profiles represented genuinely distinct learning configurations rather than arbitrary statistical partitions, mean differences on the three approaches to learning dimensions used to estimate the LPA were examined (Table 4). We can see from Table 4 that Deep–Organized students reported a significantly higher Deep approach score than Benchmark students (Δ = 0.38, *p* = 0.001), whereas Surface-Dominated students scored significantly lower (Δ = −0.67, *p* = 0.001). Deep-Dominated students did not differ significantly from the Benchmark group. Deep–Organized (Δ = −1.02, *p* < 0.001) and Deep-Dominated students (Δ = −0.84, *p* < 0.001) reported significantly lower Surface approach scores than Benchmark students, whereas Surface-Dominated students reported a significantly higher score (Δ = 0.73, *p* < 0.001). Deep–Organized students reported a significantly higher Organized approach score than Benchmark students (Δ = 0.42, *p* < 0.001), whereas Deep-Dominated (Δ = −0.93, *p* < 0.001) and Surface-Dominated (Δ = −0.83, *p* < 0.001) students reported significantly lower scores.

### 4.2 Empirical results regarding RQ2: associations between achievement goals and profile membership

Table 5 presents the Pearson (Spearman) correlations below (above) the diagonal among the study variables, offering initial insights into the relationships between achievement goals, learning profiles, and outcomes. Mastery-approach goals were positively correlated with both the proximal outcome (course total points; *r* = 0.20, *p* < 0.01) and the distal top-achievement outcome (*r* = 0.21, *p* < 0.01), while performance-approach goals displayed even stronger positive associations (proximal *r* = 0.34, *p* < 0.01; distal *r* = 0.31, *p* < 0.01). Performance-avoidance goals were also positively but weaker related to these outcomes (proximal *r* = 0.21, *p* < 0.05; distal *r* = 0.17, *p* < 0.05). Regarding profile membership, mastery and performance-approach goals correlated positively with the Deep–Organized profile (*r* = 0.33 and 0.32, respectively, *p* < 0.01) and negatively with the Surface-Dominated profile (*r* = −0.22 and −0.28, respectively, *p* < 0.01). Performance-avoidance was positively but weakly correlated with Deep–Organized membership (*r* = 0.15, *p* < 0.05) and negatively with Surface-Dominated (*r* = −0.21, *p* < 0.01).

**Table 5:** Pearson (and Spearman) correlations below (above) the diagonal (*n* = 195).

Variable	1	2	3	4	5	6	7	8	9	10	11	12	13	14
1. Proximal outcome	1.000	0.530^***^	−0.037	0.246^***^	−0.187^***^	−0.123^*^	0.254^***^	0.364^***^	0.226^***^	0.184^**^	−0.261^***^	0.103	−0.191^***^	0.296^***^
2. Distal outcome	0.348^***^	1.000	−0.032	0.130^*^	−0.118^*^	−0.043	0.205^***^	0.337^***^	0.219^***^	0.262^***^	−0.141^**^	0.155^**^	−0.189^***^	0.216^***^
3. Benchmark	−0.013	−0.032	1.000	−0.420^***^	−0.142^**^	−0.397^***^	−0.058	−0.230^***^	0.015	−0.067	0.181^**^	−0.112	0.607^***^	0.155^**^
4. Deep-Organized	0.145^**^	0.130^*^	−0.420^***^	1.000	−0.194^***^	−0.541^***^	0.332^***^	0.330^***^	0.185^***^	0.153^**^	−0.007	0.333^***^	−0.512^***^	0.672^***^
5. Surface-Dominated	−0.078	−0.118	−0.142^**^	−0.194^***^	1.000	−0.183^***^	−0.194^***^	−0.253^***^	−0.168^**^	−0.019	0.148^**^	−0.271^***^	0.403^***^	−0.205^***^
6. Deep-Dominated	−0.096	−0.043	−0.397^***^	−0.541^***^	−0.183^**^	1.000	−0.187^***^	−0.002	−0.117	−0.086	−0.231^***^	−0.100	−0.228^***^	−0.718^***^
7. Mastery-approach	0.197^***^	0.209^***^	−0.041	0.330^***^	−0.220^***^	−0.188^***^	1.000	0.406^***^	0.378^***^	−0.129^*^	−0.008	0.253^***^	−0.185^***^	0.369^***^
8. Performance-approach	0.343^***^	0.309^***^	−0.217^***^	0.323^***^	−0.284^***^	0.009	0.418^***^	1.000	0.566^***^	−0.009	−0.318^***^	0.206^***^	−0.371^***^	0.283^***^
9. Performance-avoidance	0.211^***^	0.167^**^	0.043	0.154^**^	−0.212^***^	−0.087	0.349^***^	0.616^***^	1.000	0.021	−0.059	0.111	−0.133^*^	0.269^***^
10. Matriculation points	0.106	0.239^***^	−0.062	0.117	−0.005	−0.061	−0.121^*^	−0.003	0.034	1.000	0.174^**^	0.084	−0.082	0.145^**^
11. Female	−0.172^**^	−0.141^**^	0.181^**^	−0.007	0.148^**^	−0.231^***^	−0.021	−0.320^***^	−0.095	0.128^*^	1.000	−0.128^*^	0.257^***^	0.178^**^
12. Deep approach	−0.015	0.148^**^	−0.098	0.329^***^	−0.321^***^	−0.083	0.313^***^	0.232^***^	0.087	0.011	−0.133^*^	1.000	−0.202^***^	0.220^***^
13. Surface approach	−0.118	−0.177^**^	0.539^***^	−0.493^***^	0.545^***^	−0.259^***^	−0.176^**^	−0.365^***^	−0.098	−0.086	0.263^***^	−0.236^***^	1.000	−0.120^*^
14. Organized approach	0.214^***^	0.236^***^	0.160^**^	0.643^***^	−0.213^***^	−0.689^***^	0.377^***^	0.302^***^	0.265^***^	0.131^*^	0.183^**^	0.227^***^	−0.123^*^	1.000

Pearson correlations are reported below the diagonal and Spearman rank correlations above the diagonal. *n* = 195. Female is coded 1 = female and 0 = otherwise. Benchmark, Deep-Organized, Surface-Dominated, and Deep-Dominated are profile membership indicators. ^*^p < 0.10, ^**^p < 0.05, ^***^p < 0.01.

With respect to gender, female students showed a positive correlation with membership in the Benchmark profile (*r* = 0.181, *p* < 0.05) and the Surface-Dominated profile (*r* = 0.148, *p* < 0.05). In contrast, female gender was negatively correlated with membership in the Deep-Dominated profile (*r* = −0.231, *p* < 0.01), indicating that male students were more likely to belong to this group. No statistically significant correlation emerged between female gender and the Deep–Organized profile (*r* = −0.007, non-significant).

An important distinction emerges between the bivariate associations (correlations) and the multivariate results reported below. At the bivariate level, performance-avoidance was positively associated with Deep-Organized membership and with both proximal and distal outcomes, while being negatively associated with Surface-Dominated membership. Performance-avoidance was, however, also strongly correlated with performance-approach goals (*r* = 0.616). Consequently, the regression results for performance-avoidance show its association with profile membership after accounting for performance-approach goals, mastery-approach goals, matriculation exam points, and gender. They should therefore not be interpreted as showing that performance-avoidance is generally associated with less adaptive learning.

Multicollinearity diagnostics indicated that the correlations among the predictors did not result in problematic multicollinearity. Non-tabulated VIF values ranged from 1.04 to 1.99, with the highest VIF observed for performance-approach goals (1.99), followed by performance-avoidance goals (1.67). Corresponding tolerance values ranged from 0.50 to 0.96. Thus, although performance-approach and performance-avoidance goals were substantially correlated, the multicollinearity diagnostics remained well within conventional thresholds.

Table 6 extends these analyses by estimating multinomial logistic regression models with the Benchmark profile as the reference category. Because these regressions use modal profile assignment, the estimates should be interpreted as associations with the most likely profile membership rather than with error-free latent-class membership. Both mastery (OR = 3.576, *p* = 0.01) and performance-approach goals (OR = 3.360, *p* < 0.01) significantly predicted membership in the Deep–Organized profile. Thus, students with higher mastery or performance-approach goals were more than 3 times as likely to belong to the Deep–Organized profile rather than the Benchmark profile.

**Table 6:** RQ2 examination using a multinomial regression (*n* = 195).

Membership/predictor	Log odds	Odds ratio	SE	*p-value*	Sig.
Deep-Organized					
Mastery approach	1.274	3.576	0.493	0.010	^***^
Performance approach	1.212	3.360	0.308	< 0.01	^***^
Performance avoidance	−0.833	0.435	0.312	0.008	^***^
Matriculation points	0.043	1.044	0.021	0.038	^**^
Female	−0.124	0.883	0.455	0.785	
Constant	−7.314	0.001	2.282	0.001	^***^
Surface-Dominated					
Mastery approach	−0.888	0.411	0.615	0.149	
Performance approach	−0.179	0.836	0.479	0.709	
Performance avoidance	−0.483	0.617	0.400	0.228	
Matriculation points	−0.008	0.992	0.030	0.800	
Female	0.403	1.496	0.770	0.601	
Constant	4.577	97.251	2.702	0.090	^*^
Deep-Dominated					
Mastery approach	−0.300	0.741	0.425	0.481	
Performance approach	0.762	2.142	0.291	0.009	^***^
Performance avoidance	−0.712	0.491	0.297	0.016	^**^
Matriculation points	0.010	1.010	0.019	0.616	
Female	−1.041	0.353	0.461	0.024	^**^
Constant	1.973	7.190	1.855	0.288	
Model statistics					
Mean dependent variable	2.510	SD dependent variable		1.184	
Pseudo R^2^	0.157	Number of observations		194	
Chi-square	75.973	Prob. > chi-square		< 0.01	
Akaike criterion (AIC)	443.383	Bayesian criterion (BIC)		502.205	

Benchmark is the reference membership category. Log odds are multinomial logit coefficients; odds ratios are the corresponding exponentiated coefficients. SE, standard error. Female is coded as a binary indicator. ^***^p < 0.01, ^**^p < 0.05, ^*^p < 0.10.

The results for performance-avoidance goals require more careful interpretation. The correlations reported above showed that performance-avoidance was positively related to Deep–Organized membership. However, in the regression analysis, which considers all achievement goals simultaneously, higher performance-avoidance was associated with a lower likelihood of belonging to either the Deep–Organized (OR = 0.435, *p* = 0.008) or Deep-Dominated profile (OR = 0.491, *p* = 0.016), compared with the Benchmark profile.

One possible explanation for this difference is that performance-avoidance and performance-approach goals were strongly correlated (*r* = 0.616). Students with high performance-avoidance goals also tended to have high performance-approach goals, and performance-approach goals were positively related to the deep-oriented profiles. When performance-approach and the other variables were taken into account in the regression analysis, the remaining association between performance-avoidance and the two deep-oriented profiles was negative. The low VIF values suggest that this result was not caused by problematic multicollinearity. The negative regression coefficients should therefore not be taken to mean that performance-avoidance goals are generally associated with less adaptive learning.

Matriculation exam points also increased the likelihood of Deep–Organized membership (OR = 1.04, *p* < 0.05), whereas gender was not a significant predictor.

No significant predictors emerged for the Surface-Dominated profile. However, the coefficients were negative for mastery and performance-approach, consistent with an association between lower motivation and this maladaptive orientation. The absence of significant predictors may also be attributed to the small size of this subgroup (*n* = 12). For the Deep-Dominated profile, performance-approach was a significant positive predictor (odds ratio = 2.14, *p* < 0.01). In contrast, after controlling for the other achievement goals and covariates, performance-avoidance showed a negative unique association with membership (odds ratio = 0.49, *p* < 0.05). Gender also played a role, with female students less likely to be part of this profile (odds ratio = 0.35, *p* < 0.05).

Figure 3 presents the predicted probabilities of profile membership derived from the multinomial logistic regression model reported in Table 6. The figure translates the relative risk ratios for belonging to a given profile into predicted probabilities, thereby facilitating interpretation of how mastery-approach and performance-approach goals are associated with membership in the four learning profiles.

**Figure 3:**
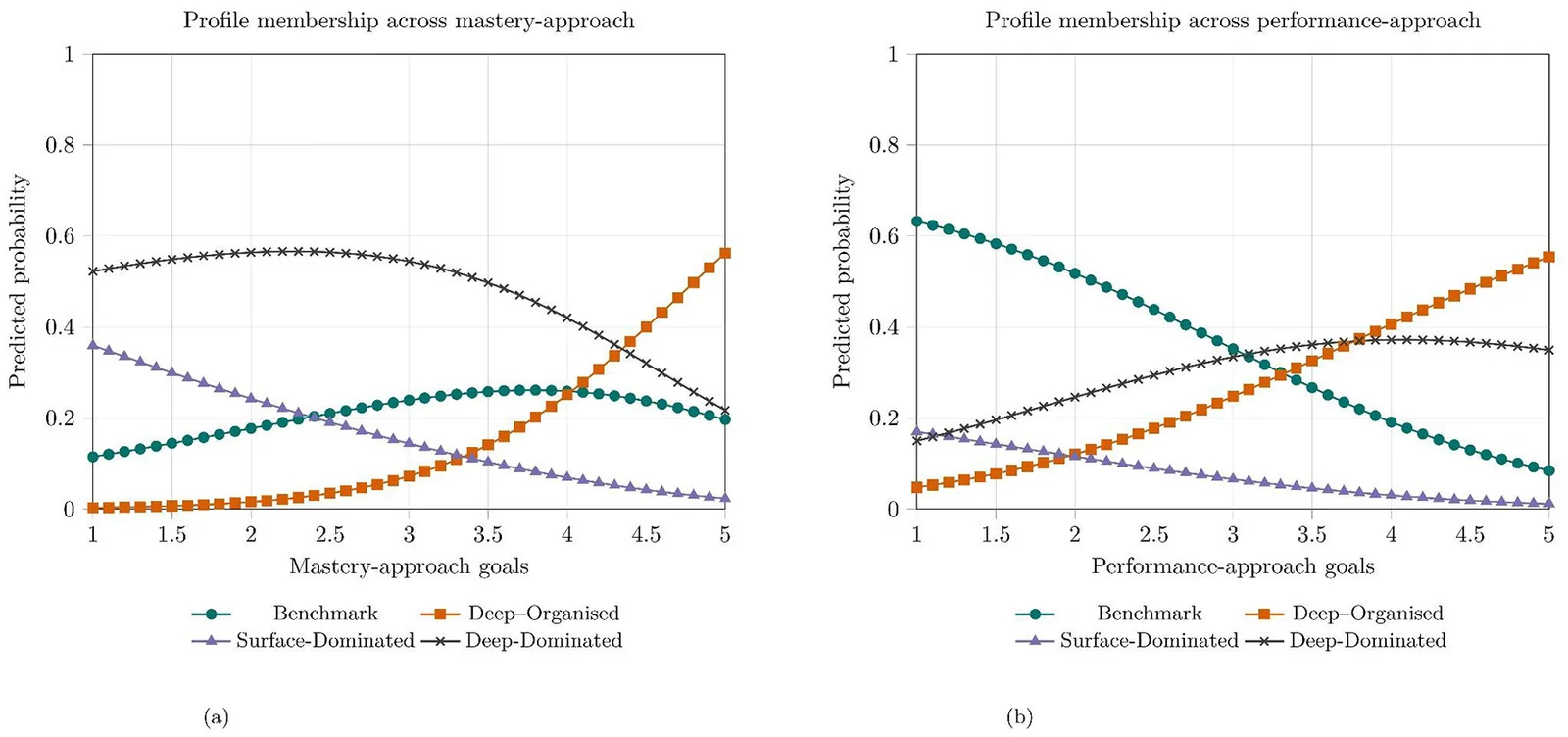
Predicted probabilities of learning-profile membership based on multinomial logit margins. **(a)** Varying mastery-approach goals. **(b)** Varying performance-approach goals.

Panel **(a)** of Figure 3 shows that as mastery-approach goals increase, the probability of belonging to the Deep–Organized profile rises clearly, while the probability of belonging to the Surface-Dominated profile declines. This visual pattern directly corresponds to the regression results in Table 6, where mastery-approach goals significantly increased the likelihood of Deep–Organized membership relative to the Benchmark profile (odds ratio = 3.58, *p* < 0.05). Mastery-approach goals were also negatively, albeit non-significantly, associated with Surface-Dominated membership. The figure further illustrates that higher mastery is associated with a gradual reduction in the probability of Deep-Dominated membership. This is consistent with the non-significant coefficient for mastery in that comparison. Panel **(a)** graphically confirms that mastery-approach orientation primarily differentiates the Deep–Organized profile from the Benchmark and Surface-Dominated profiles.

Panel **(b)** depicts the associations between performance-approach goals and predicted profile-membership probabilities. As performance-approach orientation increases, the predicted probability of belonging to both Deep–Organized and Deep-Dominated profiles increases, while the probability of Surface-Dominated membership decreases. This mirrors the regression estimates in Table 6, where performance-approach goals strongly predicted Deep–Organized membership (odds ratio = 3.36, *p* < 0.01) and significantly predicted Deep-Dominated membership (odds ratio = 2.14, *p* < 0.01). In contrast, performance-approach goals did not significantly predict Surface-Dominated membership, although the negative slope in Figure 3 reflects the direction of the coefficient. Overall, the figure complements the relative risk ratios in Table 6 by illustrating how these associations translate into changes in predicted profile probabilities across the observed range of achievement goals.

### 4.3 Empirical results regarding RQ3: the ability of AGOs and SAL profiles to predict learning outcomes

#### 4.3.1 Proximal learning outcomes

Table 7 reports the results of OLS regressions predicting students' performance in the introductory accounting course, with unstandardised coefficients and standard errors reported for each model. When learning profile membership was entered as the sole predictor (Model 1), none of the profile coefficients differed significantly from the reference (Benchmark) profile. The profile coefficients also remained non-significant after matriculation exam points and gender were added in Models 2 and 3. The coefficient for Deep–Organized was positive but non-significant, while the coefficients for Deep-Dominated and Surface-Dominated were negative and non-significant. These results indicate that students in these profiles did not differ significantly from the reference group in their course total points. Thus, learning profile membership alone was not associated with significant differences in course-level achievement.

**Table 7:** RQ3: OLS regression models for proximal learning outcomes (*n* = 195).

Predictor	(1)	(2)	(3)	(4)	(5)
Deep-Organized	3.857 (3.406)	3.414 (3.418)	2.044 (3.394)	−1.319 (3.543)	−2.368 (3.542)
Surface-Dominated	−5.076 (5.833)	−5.228 (5.824)	−4.334 (5.733)	0.064 (5.734)	0.426 (5.677)
Deep-Dominated	−2.008 (3.456)	−2.050 (3.451)	−4.470 (3.501)	−4.137 (3.460)	−5.229 (3.472)
Mastery-approach				1.535 (2.611)	2.759 (2.635)
Performance-approach				6.266^***^ (1.734)	5.406^***^ (1.799)
Performance-avoidance				−0.482 (1.713)	−0.438 (1.708)
Matriculation points		0.154 (0.120)	0.199^*^ (0.119)		0.226^*^ (0.117)
Female			−7.615^***^ (2.736)		−4.915^*^ (2.777)
Constant	79.326^***^ (2.653)	75.495^***^ (3.992)	78.524^***^ (4.071)	53.812^***^ (11.053)	48.217^***^ (11.858)
Observations	195	195	195	195	195
R^2^	0.025	0.034	0.072	0.129	0.156

The dependent variable is the proximal learning outcome, measured as total points obtained in the introductory accounting course (maximum = 106). Benchmark is the reference approaches-to-learning profile. Entries are unstandardised OLS regression coefficients; standard errors are reported in parentheses. Female is coded 1 for female students and 0 for male students. ^***^p < 0.01, ^*^p < 0.10.

By contrast, achievement goals provided more explanatory power. In Model 4, which incorporated motivational orientations, performance-approach goals emerged as a robust and statistically significant predictor of better course performance (coefficient = 6.266, *p* < 0.01). In contrast, mastery and performance-avoidance goals showed no significant associations with course performance. Female students performed significantly worse than their male counterparts (coefficient = −7.615, *p* < 0.01; Model 3).

The final model (Model 5), which included both profiles and achievement goals alongside demographic controls, explained 15.6% of the variance in course total points. In this specification, performance-approach goals remained the only consistently strong predictor (coefficient = 5.406, *p* < 0.01). The associations of mastery-approach and performance-avoidance goals with course performance remained non-significant.

#### 4.3.2 Distal learning outcomes

Table 8 presents the empirical results regarding distal learning outcomes, estimated using logistic regression models. Panel **A** reports the regression estimates, including log-odds coefficients, standard errors, odds ratios with 95% confidence intervals, and exact *p*-values; 34 students were coded as events on the distal outcome. Panel **B** reports model-level statistics for the four specifications, while Panel **C** reports average marginal effects (AMEs) for the fully adjusted model. The pattern of results largely converges with those observed for proximal outcomes and suggests that course-level performance may account for the association between learning profiles and subsequent top academic achievement. In the baseline model (Model 1) reported in Panel **A**, Deep–Organized students exhibited a marginally higher likelihood of being identified as top-performing students at the completion of the bachelor's programme (log odds = 0.830, odds ratio = 2.29, *p* < 0.10). However, this association became non-significant once course performance (the proximal outcome) was included in the model (Models 2–4). Similarly, Deep-Dominated membership did not significantly predict the distal top-achievement outcome in any specification. These findings indicate that learning profile membership alone is not a robust predictor of subsequent top academic achievement once course-level performance is considered, and they are consistent with the possibility that the association operates through proximal academic success.

**Table 8:** RQ3 Distal learning outcomes using logistic regressions (*n* = 195).

Predictor	(1)	(2)	(3)	(4)
Panel A. Logistic regression models 1–4				
Deep-Organized	*b* = 0.830^*^ SE = 0.490 OR = 2.294 95% CI = (0.878, 5.989) *p* = 0.090	*b* = −0.013 SE = 0.618 OR = 0.987 95% CI = (0.294, 3.314) *p* = 0.983	*b* = −0.074 SE = 0.644 OR = 0.928 95% CI = (0.262, 3.283) *p* = 0.908	*b* = −0.075 SE = 0.651 OR = 0.928 95% CI = (0.259, 3.323) *p* = 0.908
Deep-Dominated	*b* = 0.263 SE = 0.529 OR = 1.301 95% CI = (0.461, 3.672) *p* = 0.619	*b* = 0.434 SE = 0.656 OR = 1.543 95% CI = (0.427, 5.584) *p* = 0.508	*b* = 0.388 SE = 0.684 OR = 1.473 95% CI = (0.385, 5.633) *p* = 0.571	*b* = 0.387 SE = 0.705 OR = 1.472 95% CI = (0.370, 5.858) *p* = 0.583
Proximal outcome	–	*b* = 0.311^***^ SE = 0.060 OR = 1.365 95% CI = (1.214, 1.534) *p* = < 0.001	*b* = 0.308^***^ SE = 0.062 OR = 1.360 95% CI = (1.204, 1.537) *p* = < 0.001	*b* = 0.308^***^ SE = 0.065 OR = 1.360 95% CI = (1.198, 1.544) *p* = < 0.001
Matriculation points	–	–	*b* = 0.076^**^ SE = 0.032 OR = 1.079 95% CI = (1.013, 1.150) *p* = 0.018	*b* = 0.076^**^ SE = 0.032 OR = 1.079 95% CI = (1.013, 1.150) *p* = 0.019
Female	–	–	–	*b* = −0.002 SE = 0.581 OR = 0.998 95% CI = (0.319, 3.116) *p* = 0.997
Observations	195	195	195	195
Events	34	34	34	34
Pseudo R^2^	0.019	0.384	0.421	0.421
	**(5)**	**(6)**	**(7)**	**(8)**
Panel B. Logistic regression models 5–8				
Deep-Organized	*b* = −0.027 SE = 0.592 OR = 0.974 95% CI = (0.305, 3.104) *p* = 0.964	*b* = −0.611 SE = 0.725 OR = 0.543 95% CI = (0.131, 2.246) *p* = 0.399	*b* = −0.999 SE = 0.778 OR = 0.368 95% CI = (0.080, 1.693) *p* = 0.199	*b* = −0.998 SE = 0.778 OR = 0.369 95% CI = (0.080, 1.694) *p* = 0.200
Deep-Dominated	*b* = −0.111 SE = 0.599 OR = 0.895 95% CI = (0.277, 2.893) *p* = 0.853	*b* = 0.155 SE = 0.703 OR = 1.168 95% CI = (0.294, 4.634) *p* = 0.826	*b* = −0.132 SE = 0.755 OR = 0.876 95% CI = (0.200, 3.845) *p* = 0.861	*b* = −0.106 SE = 0.762 OR = 0.899 95% CI = (0.202, 4.001) *p* = 0.889
Mastery-approach	*b* = 0.437 SE = 0.484 OR = 1.549 95% CI = (0.599, 4.001) *p* = 0.366	*b* = 0.076 SE = 0.586 OR = 1.078 95% CI = (0.342, 3.403) *p* = 0.897	*b* = 0.826 SE = 0.667 OR = 2.284 95% CI = (0.618, 8.443) *p* = 0.216	*b* = 0.827 SE = 0.668 OR = 2.286 95% CI = (0.617, 8.470) *p* = 0.216
Performance-approach	*b* = 1.075^***^ SE = 0.363 OR = 2.929 95% CI = (1.437, 5.971) *p* = 0.003	*b* = 0.679^*^ SE = 0.399 OR = 1.972 95% CI = (0.901, 4.312) *p* = 0.089	*b* = 0.816^**^ SE = 0.399 OR = 2.260 95% CI = (1.033, 4.944) *p* = 0.041	*b* = 0.827^**^ SE = 0.401 OR = 2.287 95% CI = (1.042, 5.019) *p* = 0.039
Performance-avoidance	*b* = −0.082 SE = 0.315 OR = 0.921 95% CI = (0.497, 1.707) *p* = 0.793	*b* = 0.166 SE = 0.391 OR = 1.181 95% CI = (0.549, 2.539) *p* = 0.671	*b* = 0.024 SE = 0.388 OR = 1.024 95% CI = (0.479, 2.192) *p* = 0.951	*b* = 0.015 SE = 0.390 OR = 1.015 95% CI = (0.473, 2.181) *p* = 0.969
Proximal outcome	–	*b* = 0.295^***^ SE = 0.062 OR = 1.344 95% CI = (1.189, 1.518) *p* = < 0.001	*b* = 0.287^***^ SE = 0.067 OR = 1.333 95% CI = (1.169, 1.519) *p* = < 0.001	*b* = 0.291^***^ SE = 0.069 OR = 1.338 95% CI = (1.168, 1.533) *p* = < 0.001
Matriculation points	–	–	*b* = 0.111^***^ SE = 0.039 OR = 1.118 95% CI = (1.036, 1.206) *p* = 0.004	*b* = 0.111^***^ SE = 0.039 OR = 1.117 95% CI = (1.035, 1.205) *p* = 0.004
Female	–	–	–	*b* = 0.150 SE = 0.627 OR = 1.162 95% CI = (0.340, 3.975) *p* = 0.811
Observations	195	195	195	195
Events	34	34	34	34
Pseudo R^2^	0.134	0.409	0.470	0.471
**Variable**	**AME**	**Percentage points**	**SE**	**95% CI**	***p***
Panel C. Average marginal effects for fully adjusted Model 8					
Deep-Organized	−0.0734	−7.34	0.0535	(−0.1782, 0.0314)	0.170
Deep-Dominated	−0.0081	−0.81	0.0577	(−0.1211, 0.1050)	0.889
Mastery-approach	0.0632	6.32	0.0501	(−0.0350, 0.1615)	0.207
Performance-approach	0.0632^**^	6.32	0.0286	(0.0071, 0.1193)	0.027
Performance-avoidance	0.0012	0.12	0.0298	(−0.0573, 0.0596)	0.969
Proximal outcome	0.0223^***^	2.23	0.0039	(0.0146, 0.0300)	< 0.001
Matriculation points	0.0085^***^	0.85	0.0026	(0.0033, 0.0136)	0.001
Female	0.0115	1.15	0.0482	(−0.0830, 0.1060)	0.811

The dependent variable is the distal top-achievement outcome, coded 1 if a student was subsequently identified in publicly available business-school records as a top-performing student at the completion of the bachelor's programme and 0 otherwise.

Panels **A** and **B** report logistic regression estimates. For each predictor, b denotes the log-odds coefficient, SE its standard error, and OR the odds ratio. The 95% confidence intervals are reported for the odds ratios. Exact two-sided *p*-values are also reported. All models include an intercept, which is omitted from the table for readability.

Benchmark is the reference approaches-to-learning profile. Female is coded 1 for female students and 0 for male students.

Panel **C** reports average marginal effects (AMEs) from the fully adjusted Model 8. For continuous predictors, the AME represents the average change in the predicted probability of the distal outcome associated with a one-unit increase in the predictor. For the binary variables Deep-Organized, Deep-Dominated, and Female, the AME represents the average discrete change in predicted probability when the variable changes from 0 to 1. The percentage-points column reports 100 × AME.

Events denote the number of students coded 1 on the distal outcome in the estimation sample.

^***^p < 0.01, ^**^p < 0.05, ^*^p < 0.10.

Course performance (Proximal) emerged as a consistently strong predictor of the distal top-achievement outcome across the models (e.g., Model 2, log odds = 0.311, odds ratio = 1.365, *p* < 0.001). In other words, higher performance in the introductory accounting course was associated with a greater likelihood of subsequently being identified as a top-performing student at the completion of the bachelor's programme. Matriculation points also contributed positively to several models (e.g., Model 3, log odds = 0.076, odds ratio = 1.08, *p* < 0.05), confirming the role of prior academic preparation in the distal outcome. When motivational orientations were added (Models 5–8), performance-approach goals showed a consistently positive association with the distal outcome, reaching conventional statistical significance in Models 5, 7, and 8 and marginal significance in Model 6. Students with a stronger performance-approach orientation were more likely to be identified as top-performing students (Model 5: log odds = 1.075, odds ratio = 2.93, *p* = 0.003). Importantly, this association remained statistically significant in the fully adjusted Model 8 after controlling for course performance, matriculation exam points, and gender (log odds = 0.827, odds ratio = 2.29, *p* = 0.039).

In the Panel **C**, the marginal-effects analysis leads to the same conclusion: in the fully adjusted model, a one-unit increase in performance-approach goals was associated with an average 6.3-percentage-point increase in the predicted probability of the distal top-achievement outcome. Mastery-approach and performance-avoidance goals, however, did not significantly predict the distal top-achievement outcome. Gender also showed no significant effects in these models.

Figure 2 provides a visual summary of the study's main findings. The figure presents the results for all three research questions in an integrated manner, providing an overview of how achievement goals, learning profiles, and academic outcomes are interrelated in the context of first-semester business education. Specifically, Figure 2 illustrates the four latent learning profiles identified in our analysis and their relative positioning on the continuous Deep approach, Surface approach, and Organized approach dimensions. Second, the figure highlights the role of achievement goals in predicting students' membership in these profiles, with mastery and performance-approach goals increasing the likelihood of belonging to adaptive profiles, whereas performance-avoidance shows negative unique associations with the two deep-oriented profiles after controlling for the other achievement goals and covariates. Third, Figure 2 summarizes the predictive relationships between profiles, achievement goals, and both proximal and distal learning outcomes. In line with the regression results, the figure emphasizes that performance-approach goals emerged as the most consistent predictor of success across outcomes. At the same time, profile membership alone explained only a small amount of variance once motivational orientations were taken into account.

Figure 2 also demonstrates the extent to which the empirical results of this study interlink the three research questions: the identification of learning profiles (RQ1), the associations between achievement goals and profile membership (RQ2), and the predictive power of both goals and profiles for immediate and long-term academic success (RQ3). This synthesis underscores the central conclusion of our study. While learning profiles provide helpful descriptions of students' approaches, students' underlying motivational orientations, particularly performance-approach goals, show the strongest associations with their academic trajectories.

### 4.4 Additional empirical analysis

#### 4.4.1 A variable-oriented analysis of the association between students' approaches to learning and proximal learning outcomes

The person-oriented analyses reported above showed that approaches-to-learning profiles did not significantly predict proximal learning outcomes (Table 7). Although the Deep–Organized profile was descriptively associated with higher course performance (see Table 3; 83.18 points), profile membership alone did not explain meaningful variation in course-level achievement. This raises the possibility that aggregating learning strategies into latent profiles may obscure associations between specific dimensions of students' approaches to learning and short-term academic performance. To examine this possibility, we conducted an additional variable-oriented analysis in which the Deep, Surface, and Organized approaches were entered as continuous predictors of proximal learning outcomes. These analyses also provide a complementary robustness check because they do not depend on modal profile assignment.

The results of these analyses are presented in Table 9. As shown, the Organized approach emerged as a positive and statistically significant predictor of proximal learning outcomes (significance < 0.01 in models 1–3). Models 4 and 5 also include achievement goals and, therefore, enable a direct comparison of students' approaches to learning and achievement goals as predictors of course performance. In Models 4 and 5, the Organized approach remains positively associated with course performance, but its statistical significance weakens from *p* < 0.01 in Models 1–3 to *p* < 0.10 after achievement goals are included.

**Table 9:** Additional analysis: OLS regression models for Proximal learning outcomes using variable-oriented SAL analysis (*n* = 195).

Predictor	(1)	(2)	(3)	(4)	(5)
Deep approach	−2.671 (2.152)	−2.595 (2.153)	−3.444 (2.119)	−4.134^*^ (2.136)	−4.691^**^ (2.120)
Surface approach	−3.205 (2.070)	−3.047 (2.076)	−1.185 (2.108)	−0.343 (2.136)	0.743 (2.146)
Organized approach	5.368^***^ (1.751)	5.146^***^ (1.765)	6.465^***^ (1.771)	3.050^*^ (1.815)	3.636^*^ (1.888)
Mastery-approach				2.071 (2.644)	3.338 (2.666)
Performance-approach				5.865^***^ (1.720)	4.826^***^ (1.784)
Performance-avoidance				−0.676 (1.690)	−0.649 (1.674)
Matriculation points		0.118 (0.119)	0.167 (0.117)		0.212^*^ (0.116)
Female			−8.835^***^ (2.744)		−6.129^**^ (2.830)
Constant	79.025^***^ (11.222)	76.059^***^ (11.613)	72.864^***^ (11.380)	58.222^***^ (13.368)	51.010^***^ (13.862)
Observations	195	195	195	195	195
R^2^	0.062	0.067	0.115	0.147	0.179

The dependent variable is the proximal learning outcome, measured as total points obtained in the introductory accounting course (maximum = 106). Deep approach, Surface approach, and Organized approach are continuous Students' Approaches to Learning (SAL) measures. Entries are unstandardised OLS regression coefficients; standard errors are reported in parentheses. Female is coded 1 for female students and 0 for male students. ^***^p < 0.01, ^**^p < 0.05, ^*^p < 0.10.

#### 4.4.2 A variable-oriented analysis of the association between students' approaches to learning and distal learning outcomes

Table 10 presents the corresponding variable-oriented analysis for the distal outcome. When the three continuous SAL measures were included as predictors (Model 1), the Organized approach was positively and significantly associated with subsequent top academic achievement (*b* = 0.782, OR = 2.19, *p* = 0.009), whereas the Deep approach was not significantly associated with the outcome and the Surface approach showed a marginally significant negative association (*b* = −0.685, OR = 0.50, *p* = 0.063). Thus, students reporting stronger organized study behavior were more likely to be subsequently identified as top-performing students at the completion of the bachelor's programme. However, the association of the Organized approach became non-significant once additional predictors were introduced. In the models including achievement goals, performance-approach showed the clearest positive association with the distal outcome, although in the fully adjusted Model 8 (see panel **C**) its average marginal effect of 5.2 percentage points was only marginally significant (*p* = 0.076).

**Table 10:** Additional analysis: Distal top-achievement outcome using logistic regression and variable-oriented SAL measures (*n* = 195).

Predictor	(1)	(2)	(3)	(4)
Panel A. Logistic regression models 1–4				
Deep approach	*b* = 0.302 SE = 0.358 OR = 1.353 95% CI = (0.670, 2.731) *p* = 0.399	*b* = 0.315 SE = 0.412 OR = 1.370 95% CI = (0.612, 3.070) *p* = 0.444	*b* = 0.274 SE = 0.405 OR = 1.315 95% CI = (0.595, 2.909) *p* = 0.498	*b* = 0.277 SE = 0.420 OR = 1.319 95% CI = (0.578, 3.006) *p* = 0.510
Surface approach	*b* = −0.685^*^ SE = 0.369 OR = 0.504 95% CI = (0.245, 1.038) *p* = 0.063	*b* = −0.500 SE = 0.463 OR = 0.607 95% CI = (0.245, 1.503) *p* = 0.280	*b* = −0.401 SE = 0.470 OR = 0.669 95% CI = (0.266, 1.683) *p* = 0.393	*b* = −0.403 SE = 0.475 OR = 0.668 95% CI = (0.263, 1.697) *p* = 0.397
Organized approach	*b* = 0.782^***^ SE = 0.298 OR = 2.186 95% CI = (1.220, 3.919) *p* = 0.009	*b* = 0.285 SE = 0.374 OR = 1.329 95% CI = (0.638, 2.769) *p* = 0.447	*b* = 0.274 SE = 0.377 OR = 1.315 95% CI = (0.628, 2.754) *p* = 0.468	*b* = 0.271 SE = 0.393 OR = 1.312 95% CI = (0.607, 2.834) *p* = 0.490
Proximal outcome	–	*b* = 0.286^***^ SE = 0.059 OR = 1.331 95% CI = (1.184, 1.495) *p* = < 0.001	*b* = 0.282^***^ SE = 0.062 OR = 1.326 95% CI = (1.174, 1.497) *p* = < 0.001	*b* = 0.283^***^ SE = 0.065 OR = 1.327 95% CI = (1.169, 1.506) *p* = < 0.001
Matriculation points	–	–	*b* = 0.073^**^ SE = 0.032 OR = 1.075 95% CI = (1.010, 1.145) *p* = 0.024	*b* = 0.073^**^ SE = 0.032 OR = 1.075 95% CI = (1.009, 1.146) *p* = 0.025
Female	–	–	–	*b* = 0.014 SE = 0.620 OR = 1.014 95% CI = (0.301, 3.420) *p* = 0.982
Observations	195	195	195	195
Events	34	34	34	34
Pseudo R^2^	0.094	0.396	0.430	0.430
	**(5)**	**(6)**	**(7)**	**(8)**
Panel B. Logistic regression models 5–8				
Deep approach	*b* = 0.160 SE = 0.386 OR = 1.173 95% CI = (0.550, 2.503) *p* = 0.679	*b* = 0.338 SE = 0.431 OR = 1.402 95% CI = (0.603, 3.262) *p* = 0.433	*b* = 0.159 SE = 0.437 OR = 1.172 95% CI = (0.498, 2.761) *p* = 0.716	*b* = 0.174 SE = 0.447 OR = 1.190 95% CI = (0.496, 2.856) *p* = 0.697
Surface approach	*b* = −0.420 SE = 0.418 OR = 0.657 95% CI = (0.289, 1.491) *p* = 0.315	*b* = −0.351 SE = 0.501 OR = 0.704 95% CI = (0.264, 1.878) *p* = 0.483	*b* = −0.063 SE = 0.528 OR = 0.939 95% CI = (0.334, 2.644) *p* = 0.906	*b* = −0.070 SE = 0.530 OR = 0.933 95% CI = (0.330, 2.637) *p* = 0.896
Organized approach	*b* = 0.481 SE = 0.328 OR = 1.618 95% CI = (0.851, 3.078) *p* = 0.142	*b* = 0.080 SE = 0.390 OR = 1.083 95% CI = (0.505, 2.324) *p* = 0.838	*b* = −0.006 SE = 0.403 OR = 0.994 95% CI = (0.451, 2.192) *p* = 0.988	*b* = −0.023 SE = 0.416 OR = 0.977 95% CI = (0.432, 2.209) *p* = 0.956
Mastery–approach	*b* = 0.186 SE = 0.502 OR = 1.204 95% CI = (0.450, 3.222) *p* = 0.711	*b* = −0.163 SE = 0.607 OR = 0.849 95% CI = (0.259, 2.791) *p* = 0.788	*b* = 0.560 SE = 0.681 OR = 1.751 95% CI = (0.461, 6.657) *p* = 0.411	*b* = 0.554 SE = 0.683 OR = 1.740 95% CI = (0.456, 6.635) *p* = 0.417
Performance–approach	*b* = 0.855^**^ SE = 0.356 OR = 2.352 95% CI = (1.171, 4.727) *p* = 0.016	*b* = 0.484 SE = 0.387 OR = 1.622 95% CI = (0.760, 3.460) *p* = 0.211	*b* = 0.654^*^ SE = 0.387 OR = 1.924 95% CI = (0.901, 4.108) *p* = 0.091	*b* = 0.664^*^ SE = 0.391 OR = 1.943 95% CI = (0.903, 4.181) *p* = 0.089
Performance–avoidance	*b* = 0.004 SE = 0.320 OR = 1.004 95% CI = (0.537, 1.880) *p* = 0.989	*b* = 0.183 SE = 0.408 OR = 1.201 95% CI = (0.540, 2.673) *p* = 0.653	*b* = 0.009 SE = 0.393 OR = 1.009 95% CI = (0.467, 2.181) *p* = 0.981	*b* = 0.004 SE = 0.395 OR = 1.004 95% CI = (0.463, 2.176) *p* = 0.992
Proximal outcome	–	*b* = 0.275^***^ SE = 0.061 OR = 1.316 95% CI = (1.168, 1.483) *p* = < 0.001	*b* = 0.267^***^ SE = 0.065 OR = 1.306 95% CI = (1.150, 1.484) *p* = < 0.001	*b* = 0.270^***^ SE = 0.068 OR = 1.310 95% CI = (1.146, 1.498) *p* = < 0.001
Matriculation points	–	–	*b* = 0.100^***^ SE = 0.038 OR = 1.105 95% CI = (1.027, 1.190) *p* = 0.008	*b* = 0.100^***^ SE = 0.038 OR = 1.105 95% CI = (1.026, 1.190) *p* = 0.008
Female	–	–	–	*b* = 0.105 SE = 0.653 OR = 1.111 95% CI = (0.309, 4.000) *p* = 0.872
Observations	195	195	195	195
Events	34	34	34	34
Pseudo R^2^	0.157	0.406	0.457	0.457
**Variable**	**AME**	**Percentage points**	**SE**	**95% CI**	***p***
Panel C. Average marginal effects for fully adjusted Model 8					
Deep approach	0.0137	1.37	0.0351	(−0.0551, 0.0825)	*0.696*
Surface approach	−0.0055	−0.55	0.0417	(−0.0872, 0.0762)	*0.896*
Organized approach	−0.0018	−0.18	0.0327	(−0.0660, 0.0624)	*0.956*
Mastery–approach	0.0436	4.36	0.0533	(−0.0609, 0.1481)	*0.414*
Performance–approach	0.0523^*^	5.23	0.0295	(−0.0055, 0.1100)	*0.076*
Performance–avoidance	0.0003	0.03	0.0311	(−0.0606, 0.0612)	*0.992*
Proximal outcome	0.0213^***^	2.13	0.0042	(0.0130, 0.0296)	* < 0.001*
Matriculation points	0.0078^***^	0.78	0.0027	(0.0025, 0.0132)	*0.004*
Female	0.0083	0.83	0.0517	(−0.0930, 0.1097)	*0.872*

The dependent variable is the distal top-achievement outcome, coded 1 if a student was subsequently identified in publicly available business-school records as a top-performing student at the completion of the bachelor's programme and 0 otherwise.

Panels **A** and **B** report logistic regression estimates. Deep approach, Surface approach, and Organized approach are continuous Students' Approaches to Learning (SAL) measures. For each predictor, b denotes the log-odds coefficient, SE its standard error, and OR the odds ratio. The 95% confidence intervals are reported for the odds ratios. Exact two-sided *p*-values are also reported. All models include an intercept, which is omitted from the table for readability.

Female is coded 1 for female students and 0 for male students.

Panel **C** reports average marginal effects (AMEs) from the fully adjusted Model 8. For continuous predictors, the AME represents the average change in the predicted probability of the distal outcome associated with a one-unit increase in the predictor. For Female, the AME represents the average discrete change in predicted probability when the variable changes from 0 to 1. The percentage-points column reports 100 × AME.

Events denote the number of students coded 1 on the distal outcome in the estimation sample.

^***^p < 0.01, ^**^p < 0.05, ^*^p < 0.10.

Overall, the variable-oriented analyses suggest that the Organized approach is associated with distal academic success when learning approaches are considered alone, but this association is attenuated after additional predictors are included, whereas performance-approach goals show the more persistent positive association with the distal outcome.

## 5 Discussion and conclusions

### 5.1 Discussion

This study examined the interplay between achievement goals, students' approaches to learning, and academic outcomes in the context of first-semester business education. Three main findings emerged. First, latent profile analysis identified four distinct learning profiles, Deep–Organized, Deep-Dominated, Surface-Dominated, and Benchmark, that captured meaningful variation in students' combinations of the Deep, Surface, and Organized approaches. Second, achievement goals were associated with profile membership. Mastery and performance-approach goals were associated with an increased likelihood of belonging to adaptive profiles, whereas the findings for performance-avoidance were more nuanced. Although performance-avoidance was positively associated with Deep-Organized membership at the bivariate level, its unique association with the two deep-oriented profiles was negative after controlling for the other achievement goals and covariates. These findings align closely with the integrative evidence reported by Cano and Berbén (2009), who demonstrated substantial multivariate overlap between achievement goals and approaches to learning. In their canonical correlation and cluster analyses, mastery-approach goals were associated with a deep approach to learning, whereas performance-avoidance goals were associated with surface approaches. Moreover, distinct multiple-goal profiles differed systematically in approaches to learning and academic performance. Our results extend this work in two important ways. Whereas Cano and Berbén (2009) identified goal profiles and examined their learning consequences, we model how achievement goals associate with membership in empirically derived learning profiles. By examining both proximal and distal outcomes in a competitive business school context, the present study demonstrates that integrating AGT and SAL is not merely theoretical but predictive of long-term academic trajectories. Importantly, while Cano and Berbén (2009) showed that goal profiles differed in achievement, our results suggest that once goals are directly included in predictive models, learning profiles explain only limited additional variance. This indicates that the association between learning approaches and performance may partly reflect their shared associations with underlying motivational orientations.

Third, achievement goals, rather than learning profiles, most strongly predicted both proximal (course-level) performance and the distal indicator of top academic achievement. Across models, performance-approach goals consistently emerged as the most powerful predictor of academic success.

The identification of a Deep–Organized profile aligns with prior research by emphasizing the benefits of combining meaning-oriented engagement with systematic study management (Parpala et al., 2022). We found that students in the Deep organized profile reported the strongest mastery and performance-approach orientations, confirming that both intrinsic and extrinsic forms of motivation can underpin adaptive engagement. In contrast, the Surface-Dominated profile, though small in size, highlighted the risks of reliance on rote strategies and low motivation, consistent with evidence linking surface approach to fragmented knowledge and poorer academic performance (Everaert et al., 2017; Lucas, 2001).

Our results indicate that achievement goals are associated with adaptive learning profiles in business education. Both mastery and performance-approach orientations increase the likelihood of belonging to the Deep–Organized profile, which combines conceptual engagement with structured study habits. The Deep-Dominated profile, in contrast, is shaped mainly by performance-approach motivation, shows weaker study organization, and is more prevalent among male students. Performance-avoidance showed positive bivariate associations (correlation) with Deep-Organized membership and academic outcomes, but its associations with deep-oriented profiles became negative after adjustment. Multicollinearity diagnostics suggested that this reversal reflects a suppression or shared-variance pattern, particularly its overlap with performance-approach motivation. Thus, performance-avoidance should not be interpreted as uniformly maladaptive in this sample. The Surface-Dominated profile, finally, was not significantly explained by achievement goals, suggesting that other contextual or dispositional factors may account for its emergence.

Achievement goals played a decisive role in differentiating these profiles. Consistent with Achievement Goal Theory (Elliot and McGregor, 2001), mastery goals were positively associated with adaptive learning, whereas the role of performance-avoidance was conditional on whether its shared variance with the other achievement goals was controlled. More strikingly, performance-approach goals were associated not only with profile membership but also with both immediate and long-term academic success. These findings align with the literature review by Harackiewicz et al. (2002) on college students. In the current study, the competitive, performance-driven culture of business schools, which strives to outperform peers, may align with structured engagement and persistence, contributing to both course-level achievement and the likelihood of subsequent recognition as a top-performing student.

At the same time, the limited predictive power of learning profiles for academic outcomes, once achievement goals are included, suggests that motivational orientations may show stronger unique associations with academic success. Profiles capture patterns of study strategies, whereas underlying goals show additional associations with students' academic success over time. This highlights the value of integrating person-oriented approaches, which capture heterogeneity in learning strategies, with variable-oriented approaches that highlight the role of motivation in shaping trajectories. The additional variable-oriented analysis showed that students with higher Organized approach scores achieved higher course scores when the three approaches to learning were examined without achievement goals. In contrast, the Deep and Surface approaches did not show significant associations with proximal outcomes in these models. However, when achievement goals are entered into statistical multivariate models, the association of the Organized approach with course performance is attenuated after performance-approach goals are included.

The pattern observed here parallels the hierarchical model proposed by Diseth and Kobbeltvedt (2010), who demonstrated that achievement goals predict academic performance indirectly through learning strategies. In their structural equation model, mastery and performance-approach goals were positively associated with organized learning approaches, which in turn predicted examination grades, whereas performance-avoidance and surface approaches were negatively related to achievement. In the present study, the organized approach was initially associated with both proximal and distal outcomes, but its explanatory power diminished once achievement goals were included in the models. This pattern is consistent with the possibility that the associations between learning approaches and academic outcomes partly overlap with those of motivational orientations; however, the present cross-sectional analyses do not establish mediation or causal ordering. Our findings extend Diseth and Kobbeltvedt's (2010) results by showing that, in a highly competitive business school environment, performance-approach goals are uniquely associated with both short- and long-term academic outcomes, even after controlling for learning profiles and matriculation exam points. One possible interpretation is that performance-approach motivation may be associated with sustained engagement, persistence, and competitive orientations aligned with institutional reward structures, although these mechanisms were not directly tested in the present study.

Finally, the results revealed some gender differences consistent with prior research. Women were less likely to belong to the Deep-Dominated profile, which was strongly associated with performance-approach goals, while men were overrepresented in this profile. These findings should be regarded as exploratory, however, because they derive from a single institutional context and may reflect several factors, including prior achievement, course design, assessment format, or other unmeasured background characteristics. Although previous research has discussed the gendered nature of accounting education (Haynes, 2017), the present findings do not allow us to determine whether the observed differences reflect such contextual influences.

Taken together with the findings of Cano and Berbén (2009) and Diseth and Kobbeltvedt (2010), the present study supports the view that achievement goals and learning approaches are dynamically interconnected rather than independent constructs. Cano and Berbén (2009) highlighted their multivariate overlap and profile-based differentiation, while Diseth and Kobbeltvedt demonstrated hierarchical pathways linking goals, learning approaches, and achievement. By combining person-oriented latent profile analysis with variable-oriented regression modeling, the current study bridges these research designs and shows that performance-approach goals are consistently associated with academic success in competitive higher education contexts.

### 5.2 Conclusions, limitations, and future research

This study contributes to the literature on student motivation and learning by integrating Achievement Goal Theory (AGT) with the Student Approaches to Learning (SAL) framework in the context of business education. Using survey data from first-semester business students, we identified four distinct learning profiles that reflected different combinations of deep, surface, and organized approaches. We then examined how achievement goals were associated with membership in these profiles and how both profiles and goals predicted immediate course performance and subsequent top academic achievement during the bachelor's programme.

Three main conclusions emerge. First, students' approaches to learning are best understood through person-oriented profiles rather than single dimensions. The Deep–Organized profile, characterized by high levels of both meaning-oriented and structured study strategies, was the most adaptive, while the Surface-Dominated profile represented maladaptive engagement. Second, achievement goals proved to be important in associating with profile membership. Mastery and performance-approach orientations predicted adaptive profiles, whereas the findings for performance-avoidance were more nuanced. Overall, motivational orientations, particularly performance-approach goals, were more closely associated with academic outcomes than learning profiles, while the role of performance-avoidance was less straightforward. Performance-approach goals were consistently associated with success in the introductory accounting course and, notably, with the likelihood of subsequent recognition as a top-performing student at the completion of the bachelor's programme.

These findings advance theoretical understanding by demonstrating systematic associations between motivational orientations, students' learning profiles, and their academic outcomes. They also carry practical implications for business education. Fostering adaptive learning may require more than promoting deep and organized approaches in isolation; educators should also recognize the motivational context in which these approaches emerge. While mastery goals remain important for meaningful and sustained engagement, performance-approach goals may also play a constructive role in structured and competitive learning environments. Educational practices and interventions could therefore seek to encourage personal mastery while channeling students' competitive orientations into constructive forms of engagement.

For educators, this implies designing learning environments that support both mastery and constructive performance striving. Formative assessment, feedback-oriented teaching, and self-regulated learning opportunities may strengthen students' focus on developing competence, while appropriately structured challenges and performance standards may provide productive outlets for performance-approach motivation. From an institutional policy perspective, business schools should therefore seek an appropriate balance between competitive structures and pedagogical practices that promote meaningful learning rather than short-term achievement.

The study has some limitations. First, the sample consists of first-semester business students from a single Nordic business school, which may limit the generalisability of the findings to other academic disciplines, levels of study, or cultural contexts. For example, the observed gender differences should be considered exploratory and should not be interpreted as evidence of gender-specific motivational processes or a gendered institutional culture without replication across different courses and institutional contexts. Second, while the data capture meaningful relationships among variables, the cross-sectional design precludes causal inference. Students' approaches to learning may evolve due to various influences such as curriculum changes, personal growth, or pedagogical interventions. Third, while the study focused on achievement goals and learning approaches, it did not examine other psychological constructs, such as self-efficacy, goal commitment, or metacognitive regulation, which may further clarify the mechanisms driving student learning outcomes. Fourth, the assessment format might strengthen performance-approach orientation in accounting courses, where success is closely tied to numerical accuracy and exam performance (Huikku et al., 2022).

Fifth, the distal outcome captures only whether a student was publicly identified as a top-performing student at the completion of the bachelor's programme and therefore does not capture the full variation in students' longer-term academic performance. Individual-level cumulative GPA scores were not available from the public data source, precluding analyses using GPA as a continuous outcome. Although a continuous measure would have provided a more fine-grained assessment of longer-term academic achievement, the binary indicator nevertheless provides an independently observed distal measure of exceptional academic performance. Future research should seek to replicate the analyses using continuous cumulative GPA or other comprehensive measures of longer-term academic achievement.

Sixth, a further limitation concerns the measurement properties of the approaches-to-learning scales. Although all indicators loaded significantly on their intended latent factors and the factor correlations were theoretically coherent, the CFA yielded mixed overall fit. In particular, the CFI and TLI were below conventional benchmarks, and the Surface and Organized scales showed modest internal consistency and composite reliability. Moreover, the AVE values for all three dimensions were below 0.50. These results suggest that the measures capture the intended dimensions but also contain considerable indicator-specific variance. Findings involving the Surface and Organized approaches should therefore be interpreted cautiously, and future studies should consider further validating or refining these scales in larger and more heterogeneous samples.

Seventh, another limitation concerns uncertainty in latent-profile classification. Students were assigned to their most likely profile based on posterior membership probabilities, and these modal assignments were subsequently treated as observed categories in the regression analyses. Although classification quality was generally acceptable, with an entropy of 0.685 and class-specific average posterior probabilities ranging from 0.771 to 0.865, this approach does not explicitly correct for classification error. Consequently, associations involving profile membership may be attenuated or otherwise affected by misclassification, particularly for students whose posterior probabilities were less decisive. Future research with larger samples should apply three-step procedures that explicitly account for classification uncertainty when relating latent profiles to predictors and distal outcomes.

A further limitation concerns the timing of the data collection. The survey data were collected in Autumn 2017 and therefore reflect the institutional and pedagogical context of that period, preceding, among other developments, the substantial changes in higher education associated with the COVID-19 pandemic. Changes in course design, assessment practices, digitalisation, or institutional policies may limit the extent to which descriptive features of this cohort, such as the relative prevalence of the identified profiles, generalize to current students. However, the primary focus of the study is on theoretically grounded associations among achievement goals, approaches to learning, and academic outcomes rather than on estimating their current prevalence. Moreover, the historical cohort design enables first-semester motivational and learning characteristics to be linked with independently observed academic achievement at the completion of the bachelor's programme. Future studies should examine the replicability of these relationships in more recent cohorts and instructional contexts.

Future research should consider longitudinal designs to track changes in learning profiles across multiple academic semesters, allowing for exploring stability or transformation in motivational orientations. Expanding the scope to include qualitative data, such as student reflections or teacher interviews, could offer richer insight into how institutional culture and pedagogical practices shape learning experiences. Research could also integrate institutional variables such as course design, workload perception, and teaching quality to better understand the contextual conditions that facilitate or hinder the development of adaptive learning profiles. Finally, investigating how interventions, such as mentoring programmes, learning-skills workshops, or feedback training, can shift students from maladaptive to more productive learning profiles may provide practical guidance for improving academic outcomes in business education and beyond.

An important avenue for future research is to examine whether the relationships observed here generalize beyond business education. We would expect some features, such as the benefits associated with deep and organized approaches to learning, to generalize across disciplinary contexts. However, the role of achievement goals may be more context-dependent. In particular, the positive association of performance-approach goals with academic outcomes observed in this study may be especially salient in structured and competitively assessed environments such as business education. In disciplines characterized by different assessment practices, greater collaboration, or less emphasis on relative performance, mastery-approach goals may play a comparatively stronger role. Cross-disciplinary replication would therefore help distinguish general motivational processes from associations that are specific to particular educational contexts.

## Data Availability

The datasets presented in this article are not readily available because the datasets generated and analyzed during the current study are not publicly available due to ethical restrictions. Specifically, the data were collected under informed consent agreements that did not include permission for public data sharing. As a result, sharing the data would violate the conditions under which participants consented to participate. Requests to access the datasets should be directed to hannu.ojala@uef.fi.

## Appendix. Variable definitions

**Table A1:**

Variable	Description
*Deep approach*	Mean score measuring the deep approach to learning using the LEARN questionnaire (Parpala and Lindblom-Ylänne, 2012).^a)^
*Surface approach*	Mean score measuring the surface approach to learning using the LEARN questionnaire (Parpala and Lindblom-Ylänne, 2012).^a)^
*Organized approach*	Mean score measuring the organized approach to learning using the LEARN questionnaire (Parpala and Lindblom-Ylänne, 2012).^a)^
*Deep-Organized*	Students' learning profile based on the latent profile analysis.
*Deep-Dominated*	Students' learning profile based on the latent profile analysis.
*Benchmark*	Students' learning profile based on the latent profile analysis.
*Surface-Dominated*	Students' learning profile based on the latent profile analysis.
*Mastery approach*	Mean of the raw scores constructed from ‘Achievement Goal Questionnaire revised' (AGQ-revised) by (Elliot and Murayama 2008).
*Performance approach*	Mean of the raw scores constructed from ‘Achievement Goal Questionnaire revised' (AGQ-revised) by (Elliot and Murayama 2008).
*Performance avoidance*	Mean of the raw scores constructed from ‘Achievement Goal Questionnaire revised' (AGQ-revised) by (Elliot and Murayama 2008).
*Proximal learning outcome*	The total points for the Introduction to Accounting course (maximum 106 points), consisting of the final course exam (max. 60 points), two open-book mid-term exams (max. 20 points) and teamwork points (max. 26 points), to indicate immediate academic performance
*Distal learning outcome*	Binary indicator of top academic achievement during the bachelor's programme. Equals 1 if the student was identified in publicly available business-school records as a top-performing student at the completion of the bachelor's programme, and 0 otherwise. The measure was not constructed by dichotomising individual-level cumulative GPA data; continuous GPA scores were not available from the public data source.
*Female*	Equals 1 if the student is female and zero if the student is male.
*Matriculation points*	High school grade point average.

^a)^ Original version: The students' Approaches to Learning and Studying Inventory (ALSI) (Entwistle and McCune, 2004) and Experiences of Teaching and Learning Questionnaire (ETLQ) (Parpala et al., 2010).

## References

[B1] Ames C. (1992). Classrooms: goals, structures, and student motivation. J. Educ. Psychol. 84, 261–271. doi: 10.1037/0022-0663.84.3.261

[B2] Biggs J. B. (1987). Student Approaches to Learning and Studying. Hawthorn: Australian Council for Educational Research.

[B3] Bråten I. Strømsø H. I. (2006). Predicting achievement goals in two different academic contexts: a longitudinal study. Scand. J. Educ. Res. 50, 127–148. doi: 10.1080/00313830600575932

[B4] Byrne M. Flood B. (2007). Exploring the antecedents of learning approaches: a study of international business students. Int. J. Manag. 6, 44–62. doi: 10.3794/ijme.62.197

[B5] Cano F. Berbén A. B. G. (2009). University students' achievement goals and approaches to learning in mathematics. Br. J. Educ. Psychol. 79, 131–153. doi: 10.1348/000709908X314928 18578956

[B6] Collins L. M. Lanza S. T. (2013). Latent Class and Latent Transition Analysis: With Applications in the Social, Behavioral, and Health Sciences. Hoboken, NJ: John Wiley and Sons.

[B7] DeShon R. P. Gillespie J. Z. (2005). A motivated action theory account of goal orientation. J. Appl. Psychol. 90, 1096–1127. doi: 10.1037/0021-9010.90.6.1096 16316268

[B8] Diseth A. Kobbeltvedt T. (2010). A mediation analysis of achievement motives, goals, learning strategies, and academic achievement. *Br. J. Educ. Psychol*. 80, 671–687. doi: 10.1348/000709910X492432 20470452

[B9] Dweck C. S. Leggett E. L. (1988). A social-cognitive approach to motivation and personality. Psychol. Rev. 95, 256–273. doi: 10.1037/0033-295X.95.2.256

[B10] Elliot A. J. (1999). Approach and avoidance motivation and achievement goals. Educ. Psychol. 34, 169–189. doi: 10.1207/s15326985ep3403_3

[B11] Elliot A. J. Church M. A. (1997). A hierarchical model of approach and avoidance achievement motivation. J. Person. Soc. Psychol. 72, 218–232. doi: 10.1037/0022-3514.72.1.218 10234849

[B12] Elliot A. J. McGregor H. A. (2001). A 2 × 2 achievement goal framework. J. Person. Soc. Psychol. 80, 501–519. doi: 10.1037/0022-3514.80.3.501 11300582

[B13] Elliot A. J. Murayama K. (2008). On the measurement of achievement goals: critique, illustration, and application. J. Educ. Psychol. 100, 613–628. doi: 10.1037/0022-0663.100.3.613

[B14] Entwistle N. McCune V. (2004). The conceptual bases of study strategy inventories. Educ. Psychol. Rev. 16:4, 325–345. doi: 10.1007/s10648-004-0003-0

[B15] Entwistle N. Ramsden P. (1983). Understanding Student Learning. London: Croom Helm.

[B16] Everaert P. Opdecam E. Maussen S. (2017). The relationship between motivation, learning approaches, academic performance, and time spent. Account. Educ. 26, 78–107. doi: 10.1080/09639284.2016.1274911

[B17] Fenollar P. Román S. Cuestas P. J. (2007). University students' academic performance: an integrative conceptual framework and empirical analysis. Br. J. Educ. Psychol. 77, 873–891. doi: 10.1348/000709907X189118 17535509

[B18] Gow L. Kember D. Cooper B. (1994). The teaching context and approaches to study of accounting students. Issues Account. Educ. 9, 118–130. doi: 10.2308/IAE-9605235153

[B19] Harackiewicz J. M. Barron K. E. Carter S. M. Lehto A. T. Elliot A. J. (1997). Predictors and consequences of achievement goals in the college classroom: maintaining interest and making the grade. J. Person. Soc. Psychol. 73, 1284–1295. doi: 10.1037//0022-3514.73.6.1284

[B20] Harackiewicz J. M. Barron K. E. Pintrich P. R. Elliot A. J. Thrash T. M. (2002). Revision of achievement goal theory: necessary and illuminating, J. Educ. Psychol. 94, 638–645. doi: 10.1037/0022-0663.94.3.638

[B21] Haynes K. (2017). Accounting as gendering and gendered: a review of 25 years of critical accounting research on gender. Crit. Perspect. Account. 43, 110–124. doi: 10.1016/j.cpa.2016.06.004

[B22] Huikku J. Myllymäki E.-R. Ojala H. (2022). Gender differences in the first course in accounting: an achievement goal approach. Br. Account. Rev. 54:101081. doi: 10.1016/j.bar.2022.101081

[B23] Huikku J. Ojala H. Mättö M. (2026). Understanding the shift: exploring motivational and contextual factors affecting the adoption of a learning approach. Account. Educ. 35, 526–562. doi: 10.1080/09639284.2025.2483835

[B24] Lucas U. (2001). Deep and surface approaches to learning within introductory accounting: a phenomenographic study. Account. Educ. 10, 161–184. doi: 10.1080/09639280110073443

[B25] Lucas U. Tan P. L. (2013). Developing a reflective capacity within undergraduate education: the role of work-based placement learning. Assess. Eval. High. Educ. 38, 537–554. doi: 10.1080/03075079.2011.569706

[B26] Marsh H. W. Lüdtke O. Trautwein U. Morin A. J. (2009). Classical latent profile analysis of academic self-concept dimensions: synergy of person-and variable-centered approaches to theoretical models of self-concept. Struct. Equ. Modeling 16, 191–225. doi: 10.1080/10705510902751010

[B27] Marton F. Säljö R. (1976). On qualitative differences in learning: i—Outcome and process. Br. J. Educ. Psychol. 46, 4–11. doi: 10.1111/j.2044-8279.1976.tb02980.x

[B28] Meece J. L. Glienke B. B. Burg S. (2006). Gender and motivation. J. Sch. Psychol. 44, 351–373. doi: 10.1016/j.jsp.2006.04.004

[B29] Morin A. J. Morizot J. Boudrias J. S. Madore I. (2011). A multifoci person-centered perspective on workplace affective commitment: a latent profile/factor mixture analysis. Organ. Res. Methods 14, 58–90. doi: 10.1177/1094428109356476

[B30] Niemivirta M. Pulkka A. T. Tapola A. Tuominen H. (2019). “Achievement goal orientations: a person-oriented approach,” in The Cambridge handbook of motivation and learning (Cambridge: Cambridge University Press), 566–616. doi: 10.1017/9781316823279.025

[B31] Nylund K. L. Asparouhov T. Muthén B. O. (2007). Deciding on the number of classes in latent class analysis and growth mixture modeling: a Monte Carlo simulation study. Struct. Equ. Modeling 14, 535–569. doi: 10.1080/10705510701575396

[B32] Parpala A. Lindblom-Ylänne S. (2012). Using a research instrument for developing quality at the university. Qual. High. Educ. 18, 313–328. doi: 10.1080/13538322.2012.733493

[B33] Parpala A. Lindblom-Ylänne S. Komulainen E. Litmanen T. Hirsto L. (2010). Students' approaches to learning and their experiences of the teaching–learning environment in different disciplines. Br. J. Educ. Psychol. 80, 269–282. doi: 10.1348/000709909X476946 19906328

[B34] Parpala A. Ruohoniemi M. Karvonen J. Lindblom-Ylänne S. (2022). Approaches to learning and their impact on academic achievement: exploring learning profiles in higher education, Stud. High. Educ. 47, 1671–1685. doi: 10.1080/00313831.2021.1958256

[B35] Pintrich P. R. (2000). Multiple goals, multiple pathways: The role of goal orientation in learning and achievement. J. Educ. Psychol. 92, 544–555. doi: 10.1037/0022-0663.92.3.544

[B36] Puhakka I. J. Niemivirta M. Postareff L. Nokelainen P. (2025). A person-centered study of construction sector workers' goal orientation profiles and their connections to approaches to learning, self-efficacy, and work engagement. Vocat. Learn. 18:4. doi: 10.1007/s12186-025-09362-9

[B37] Struyven K. Dochy F. Janssens S. (2005). Students' perceptions about evaluation and assessment in higher education: a review. Assess. Eval. High. Educ. 30, 325–341. doi: 10.1080/02602930500099102

[B38] Tein J. Y. Coxe S. Cham H. (2013). Statistical power to detect the correct number of classes in latent profile analysis. Struct. Equ. Modeling 20, 640–657. doi: 10.1080/10705511.2013.824781 PMC3904803 24489457

[B39] Tempelaar D. T. Rienties B. Giesbers B. Gijselaers W. H. (2015). The pivotal role of effort beliefs in mediating implicit theories of intelligence and achievement goals and academic motivations. Soc. Psychol. Educ. 18, 101–120. doi: 10.1007/s11218-014-9281-7

